# High Connectivity at Abyssal Depths: Genomic and Proteomic Insights Into Population Structure of the Pan‐Atlantic Deep‐Sea Bivalve *Ledella ultima* (E. A. Smith, 1885)

**DOI:** 10.1002/ece3.71903

**Published:** 2025-08-08

**Authors:** Jenny Neuhaus, Mark E. de Wilt, Sven Rossel, Saskia Brix, Ron J. Etter, Robert M. Jennings, Katrin Linse, Pedro Martínez Arbizu, Martin Schwentner, Janna Peters

**Affiliations:** ^1^ Senckenberg am Meer, German Centre for Marine Biodiversity Research (DZMB) Hamburg Germany; ^2^ Institute of Marine Ecosystem and Fishery Science, Benthic Biogeography University of Hamburg Hamburg Germany; ^3^ Faculty of Science and Engineering University of Groningen Groningen the Netherlands; ^4^ Ifremer, BEEP, Univ Brest Plouzané France; ^5^ Senckenberg am Meer, German Centre for Marine Biodiversity Research (DZMB) Wilhelmshaven Germany; ^6^ Biology Department University of Massachusetts Boston Boston Massachusetts USA; ^7^ Biology Department Temple University Philadelphia Pennsylvania USA; ^8^ British Antarctic Survey Cambridge UK; ^9^ Natural History Museum Vienna, 3. Zoology Vienna Austria

**Keywords:** 2b‐RAD, DNA barcoding, MALDI‐TOF MS, mitochondrial heteroplasmy, phylogeography

## Abstract

Phylogeographic analyses have advanced our understanding of evolutionary processes in the deep sea, yet patterns of genetic variation and population divergence at abyssal depths remain poorly understood. The bivalve *Ledella ultima* is one of the most abundant protobranchs in the abyssal Atlantic, making it a valuable model organism for studying phylogeographic patterns and population connectivity. However, evidence for sex‐specific heteroplasmic mtDNA challenges the assessment of genetic structure using mitochondrial markers alone. To address this, we used mtDNA (COI, 16S), single‐nucleotide polymorphisms (SNPs) from 2b‐RAD, and proteomic profiles to examine the population structure of *L. ultima* across seven Atlantic basins spanning over 10,000 km in latitude. Five mitochondrial lineages with a lack of geographic structure were consistently identified by COI and 16S. Conversely, SNP and proteomic data did not mirror these findings, denoting that heteroplasmic mtDNA inflates intraspecific genetic divergence in this gonochoric species. Despite the SNP data revealing overall low genetic divergence, subtle genetic structure was detected by admixture analyses supporting two source populations: one in the north and central Atlantic, and a second in the south Atlantic, with moderate admixture in the Brazil and Cape basins. Proteomic fingerprinting revealed two basin‐separated groups with patterns distinct from the nuclear data, suggesting environmentally driven shifts in protein expression. Our findings underscore the value of integrating nuclear genomic and proteomic tools to decipher population connectivity at abyssal depths, where minimal genetic differentiation necessitates fine‐scale analyses.

## 1 Introduction

Phylogeographic patterns in the deep sea are shaped by a complex interplay of oceanographic dynamics, dispersal mechanisms, and evolutionary processes (Gage and Tyler 1991; Ramirez‐Llodra et al. 2010; Rex and Etter 2010). While connectivity studies along seamounts, hydrothermal vents, and mid‐ocean ridges have been a major focus in recent years (Boschen‐Rose and Colaço 2021; Breusing et al. 2016; Portanier et al. 2023; Yearsley et al. 2020), the vast abyssal plains, covering more than half of the Earth's surface, remain largely unexplored in this context (reviewed in Baco et al. 2016; Taylor and Roterman 2017). To counteract these knowledge gaps, large international initiatives, such as the Census of Marine Life (CoML; Ausubel et al. 2010), the Deep Ocean Steward Initiative (DOSI; https://www.dosi‐project.org), and the UN Ocean Decade (https://oceandecade.org) were launched. Assigned to the program Challenger 150 (https://challenger150.world/), the action IceDivA (Icelandic marine Animals meets Diversity along latitudinal gradients in the deep sea of the Atlantic Ocean; Brix and Taylor 2021) served as a knowledge hub by revisiting fundamental hypotheses in deep‐sea science and investigating connectivity patterns along latitudinal gradients on a pan‐Atlantic scale. Building upon previous expeditions run in the frame of the CoML, samples collected over the last decades are available as a unique pan‐Atlantic dataset (Kürzel et al. 2023).

Protobranch bivalves rank among the most abundant invertebrates in the deep Atlantic (Allen 2008; Gage and Tyler 1991; Zardus 2002), contribute to the ecological complexity of soft‐bottom habitats (Ellingsen et al. 2007; Reed et al. 2014), and have proven to be valuable model organisms to investigate phylogeographic patterns, genetic diversity, and evolutionary processes (Chase et al. 1998; Etter et al. 1999, 2005; Zardus et al. 2006; Jennings et al. 2013). In these studies, abyssal populations were consistently found to exhibit less genetic divergence when compared to bathyal populations at a comparable geographic scale, supportive of the depth‐differentiation hypothesis (DDH) (Etter et al. 2005; Etter and Rex 1990; Rex and Etter 2010). Similar patterns of bathymetric zonation and genetic diversity have been found in other marine taxa, disclosing genetic barriers across depth strata (Howell et al. 2002; Quattrini et al. 2015; Zhang et al. 2021). Although the DDH highlights a general trend of decreasing population differentiation with depth, processes that govern genetic divergence and connectivity of species at abyssal depths (3500–6000 m) are far from understood (Gary et al. 2020; Riehl et al. 2020; Rogers and Ramirez‐Llodra 2024). Precisely because intraspecific genetic differentiation appears inconspicuous, mitochondrial gene markers might not have the resolution needed to capture subtle patterns of genetic structure and connectivity in abyssal environments. Allowing for the identification of thousands of genome‐wide single‐nucleotide polymorphisms (SNPs) from a high number of individuals (Andrews et al. 2016; Davey and Blaxter 2010), the use of restriction site‐associated DNA sequencing (RAD‐seq) provides advanced assessments of fine‐scale population structure in marine species (Benestan et al. 2015; Galaska et al. 2017; Reitzel et al. 2013). To complement DNA‐based population genetic analyses herein, we used proteomic fingerprinting, which has been increasingly applied to assess phenotypic consistency and to explore whether molecular divergence is reflected at the protein expression level (Peters et al. 2023; Renz et al. 2021; Rossel et al. 2024). Proteomic fingerprinting is based on the mass detection of peptide and low molecular weight protein molecules and is used for microbial species identification (Singhal et al. 2015) and has also proven applicable to a wide range of metazoan taxa (Halada et al. 2018; Karger et al. 2019; Yssouf et al. 2014).

The small protobranch *Ledella ultima* is a true abyssal species and abundant at depths of 3000–5800 m. Spanning from the British continental margin to the Agulhas Basin in the east, and from the North American margin to the Argentine and Scotia seas in the west (Allen 2008; Allen and Hannah 1989; Allen and Sanders 1996; Clarke 1961; Janssen and Krylova 2014), the species has a pan‐Atlantic distribution and serves as a valuable model for studying phylogeographic patterns and population connectivity (Etter et al. 2005, 2011). Within a population, females and males are inferred to occur in approximately equal numbers, supported by the commonly observed balanced sex ratio in protobranchs (Zardus 2002) and the documented sex ratios in the Atlantic sister species *L. pustulosa* and *L. sublevis* (Allen and Hannah 1989). Populations of *L. ultima* are suggested to follow a continuous breeding pattern with the development of planktonic lecithotrophic pericalymma larvae that passively disperse by bottom currents (Gage and Tyler 1991; Tyler et al. 1992; Zardus and Morse 1998; Zardus 2002; Young 2003). The bivalve can grow to a maximum of 3.4 mm in shell length. Smallest specimens of females and males with developing gonads were reported with respective shell lengths of 2.4 and 2.5 mm, where females may develop 9–29 ova of 160–255 μm in diameter and a subsequent larval shell length of 310 μm (Allen and Hannah 1989).

**FIGURE 1:**
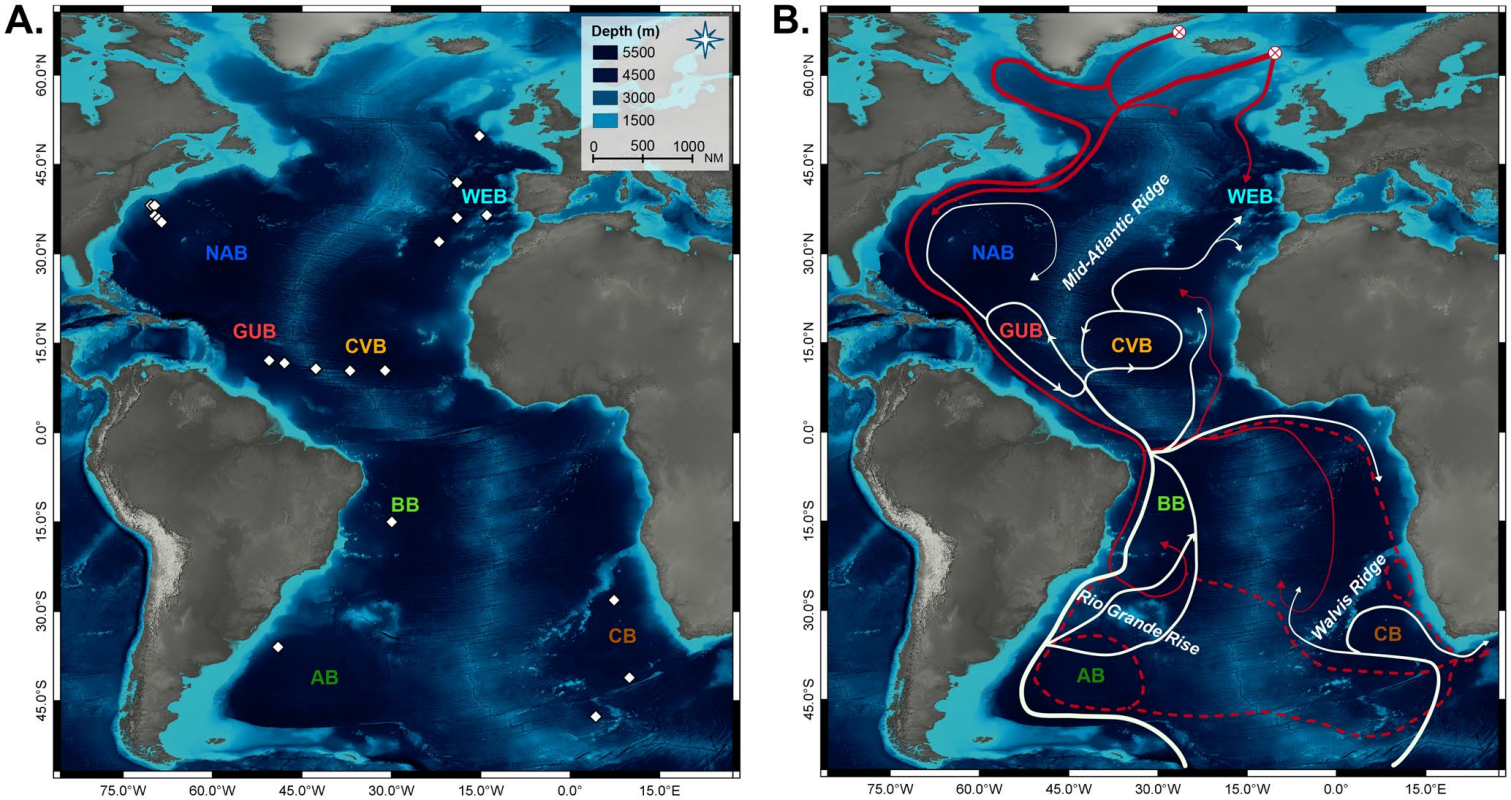
Atlantic deep‐sea basins where specimens of *L. ultima* were collected. (A) Sampled stations and bathymetric profile of the North American Basin (NAB), Guyana Basin (GUB), Brazil Basin (BB), Argentine Basin (AB), Cape Basin (CB), Cape Verde Basin (CVB), and West European Basin (WEB). Fully overlapping stations are displayed as a single shape. (B) Qualitative deep‐water circulation patterns of Antarctic Bottom Water (AABW, beige) and North Atlantic Deep Water (NADW, red). Stippled lines show NADW propagating above 3500‐m depth. Crossed circles indicate NADW formation areas. Water mass directions are derived from Morozov et al. (2010), Garzoli and Matano (2011), and Ferreira and Kerr (2017). Underlying bathymetry provided by GEBCO 2020 Grid. Map drawn in WGS84.

The main deep‐ocean basins where *L. ultima* is found are the Western European Basin (WEB), North American Basin (NAB), Brazil Basin (BB), Argentine Basin (AB), and Cape Basin (CB) (Figure 1A; Warén 1981; Filatova and Schileyko 1984; Allen and Hannah 1989; Allen and Sanders 1996; Allen 2008; Etter et al. 2011). In these basins, large‐scale oceanographic processes of North Atlantic Deep Water (NADW) and Antarctic Bottom Water (AABW) play a crucial role in shaping benthic biodiversity patterns by influencing the dispersal of deep‐water fauna (Jollivet et al. 2024; Kuhlbrodt et al. 2007; Puerta et al. 2020; Talley 2013). Formed over the continental slope of Antarctica, AABW is the densest water mass and occupies almost the entire bottom layer of the Atlantic, whereas NADW, formed in the North Atlantic, is found between 2000 and 4000‐m depth and is distinguished from Antarctic waters by higher temperature and salinity, and a lower concentration of nutrients (Dickson and Brown 1994; Ferreira and Kerr 2017; Liu and Tanhua 2021; Watling et al. 2013). These water masses propagate through the abyssal basins, many of which are confined by topographic features such as the Mid‐Atlantic Ridge (MAR) separating eastern and western Atlantic corridors, the Rio Grande Rise separating the AB and BB in the south‐west, and the Walvis Ridge confining the CB to the north (Morozov et al. 2010) (Figure 1B). For AABW and NADW to overcome these barriers, trenches and fracture zones serve as main pathways for bottom‐water exchange and connectivity between basins (Morozov et al. 2010, 2023). Since most benthic deep‐sea species rely on passive transport processes for dispersal (Etter and Bower 2015; Hilário et al. 2015; Ross et al. 2020), basin connectivity is of fundamental importance for understanding phylogeographic patterns and population connectivity at large geographic scales.

Other than in most taxa, where mitochondrial DNA (mtDNA) is homoplasmic and transmitted through strict maternal inheritance (Birky 2001), Boyle and Etter (2013) provided evidence for *L. ultima* to exhibit mitochondrial heteroplasmy. Owed to the simultaneous presence of two or more types of mtDNA in both sexes, mitochondrial heteroplasmy can result in high intraspecific genetic divergence and challenge the design of population genetic analyses based on mitochondrial markers alone (Martínez et al. 2023; Rodríguez‐Pena et al. 2020; Wai Ho and Hanafiah 2024). To overcome this, we applied a high‐resolution SNP‐based approach to identify fine‐scale population structure beyond the resolution of the mtDNA gene markers COI and 16S, as well as an assessment of proteome fingerprints to aid the analysis of genetic differentiation and investigate potential ecological subdivisions within the species.

## 2 Materials and Methods

### 2.1 Sampling Design

**FIGURE 2:**
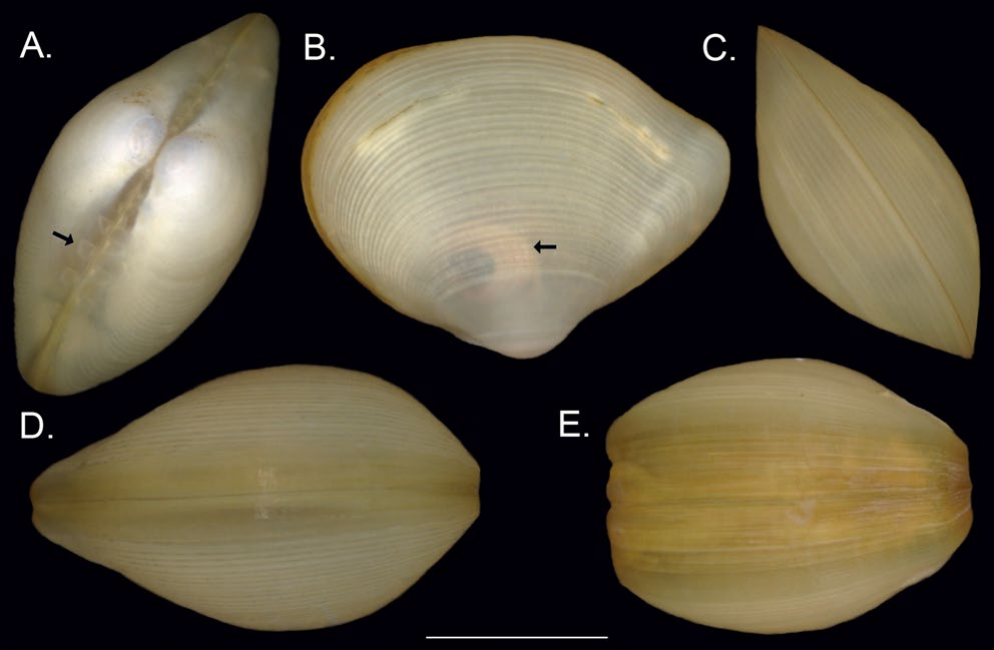
External morphology of *L. ultima* . (A) Dorsal view with interlocking hinge teeth (black arrow; (Biv_321)) (B) Left valve of a translucent shell with a visible multiple coiled gut (black arrow; Biv_218). (C–E) Ventral view of specimens with levels of edge thickening: None (Biv_216), present (Biv_178), and pronounced (Biv_183). Scale bar: 1 mm.

Specimens of *L. ultima* were collected during seven research expeditions between 2005 and 2021 (see Table 1). The majority of samples were obtained with an epibenthic sledge (EBS; Brenke 2005), while 29 specimens were sampled either by Agassiz trawl (AGT), box corer (BC), or remotely operated vehicle (ROV). Specimens were identified to species level using a Leica MZ8 stereomicroscope and taxonomic literature (Allen and Hannah 1989; Filatova and Schileyko 1984; Knudsen 1970; Smith 1885). The species is characterized by a robust shell with prominent concentric growth lines (Figure 2) and can reach up to 3.4 mm in shell length. Like many protobranchs, a strong hinge‐teeth mechanism is present on each side of the ligament (Figure 2A). *Ledella ultima* differs from all other Atlantic species by its tightly coiled hind gut situated at the dorsal margin (Figure 2B). Not unique, but by far the most pronounced, is a thickening of the ventral shell edge which occurs in some but not all specimens that have reached a shell length of 2.4 mm or more (Allen and Hannah 1989; Figure 2D–E). Shells from a maximum of five specimens per station were photographed using a Keyence VHX‐7000 digital microscope. Images are deposited under accession doi: 10.5883/DS‐LEDUL. All specimens were stored in 96% ethanol and kept at 4°C–8°C to facilitate DNA extraction and amplification.

**TABLE 1:** Station data and number (*n*) of *L. ultima* specimens collected during seven research expeditions in the North American Basin (NAB), Guyana Basin (GUB), Brazil Basin (BB), Argentine Basin (AB), Cape Basin (CB), Cape Verde Basin (CVB), and West European Basin (WEB).

Station	Year	Depth (m)	Latitude	Longitude	Basin	Gear	*n*	References
ANDEEP III (PS67)								Fahrbach (2006)
16–10	2005	4687	41°07.06′ S	009°54.88′ E	CB	EBS	2	
16–11	2005	4699	41°07.46′ S	009°55.11′ E	CB	AGT	13	
21–7	2005	4555	47°38.73′ S	004°15.20′ E	CB	EBS	3	
21–8	2005	4578	47°39.19′ S	004°16.50′ E	CB	AGT	13	
DIVA 2 (M63/2)								Türkay and Pätzold (2009)
26	2005	5040	28°06.65′ S	007°20.85′ E	CB	BC	1	
ENAB^a^ (EN477)								Etter and Rex (2021)
17	2008	3500	38°10.41′ N	70°18.13′ W	NAB	EBS	4	
17a	2008	3500	38°08.00′ N	70°19.00′ W	NAB	EBS	2	
18a	2008	3800	38°05.59′ N	69°42.45′ W	NAB	EBS	12	
20	2008	4400	36°21.03′ N	69°41.30′ W	NAB	EBS	24	
21	2008	4700	35°52.12′ N	69°03.58′ W	NAB	EBS	26	
22	2008	5000	35°18.30′ N	68°32.94′ W	NAB	EBS	6	
DIVA 3 (M79/1)								Martínez Arbizu et al. (2009)
532	2009	4605	35°59.16′ S	049°00.75′ W	AB	EBS	6	
579	2009	5182	14°58.41′ S	029°57.51′ W	BB	EBS	14	
580	2009	5131	14°58.91′ S	029°56.49′ W	BB	EBS	23	
Vema‐TRANSIT (SO237)								Devey (2015)
4‐8	2014	5735	10°24.161′ N	31°06.205′ W	CVB	EBS	22	
4‐9	2014	5733	10°24.589′ N	31°04.247′ W	CVB	EBS	34	
6‐7	2015	5085	10°20.659′ N	36°57.010′ W	CVB	EBS	1	
6‐8	2015	5127	10°22.293′ N	36°55.852′ W	CVB	EBS	2	
8‐4	2015	5178	10°43.000′ N	42°39.723′ W	VFZ	EBS	1	
9‐8	2015	5004	11°39.014′ N	47°56.168′ W	GUB	EBS	5	
11‐1	2015	5093	12°05.732′ N	50°30.239′ W	GUB	EBS	4	
14‐2	2015	4925	19°03.877′ N	67°08.100′ W	PRT	EBS	3	
IceAGE 3 (SO276)								Brix et al. (2020)
133‐8	2020	4621	49°48.031′ N	015°13.004′ W	WEB	ROV	1	
138‐1	2020	4570	49°47.352′ N	015°14.553′ W	WEB	BC	1	
IceDivA 1 (SO280)								Brix and Taylor (2021)
21‐1	2021	4802	41°57.599′ N	018°58.832′ W	WEB	EBS	14	
28‐1	2021	4904	41°57.554′ N	018°58.768′ W	WEB	EBS	15	
40‐1	2021	5484	36°02.328′ N	018°59.497′ W	WEB	EBS	4	
61‐1	2021	5121	32°02.025′ N	022°00.652′ W	WEB	EBS	3	
85‐1	2021	4155	36°28.803′ N	013°59.617′ W	WEB	EBS	2	

*Note:* In addition, specimens were collected from the Vema Fracture Zone (VFZ) and the Puerto Rico Trench (PRT).

Abbreviations: AGT, Agassiz trawl; BC, box corer; EBS, epibenthic sledge; ROV, remotely operated vehicle.

^a^ Except for six specimens from station 21, ENAB samples were only accessible as COI sequences.

### 2.2 Molecular Analyses

#### 2.2.1 Tissue Preparation

Assessments of the internal morphology and sexing of the specimens were not performed, leaving the count of females and males unknown. Tissue samples for molecular and proteomic works were prepared concurrently using sterile utensils. For each specimen, complete soft tissue (somatic and gonad tissue) was taken, where possible excluding the gut. Soft tissue fractions of each individual were stored in 96% ethanol prior to further processing for DNA extraction and proteomic fingerprinting.

#### 2.2.2 Mitochondrial Markers

DNA was extracted using the E.Z.N.A Mollusk DNA kit (Omega Bio‐Tek Inc., Norcross, GA, USA), following the manufacturer's instructions and leaving the tissue for digestion overnight in a shaking bath at 56°C/350 rpm. For all isolates, elution was carried out in two steps, applying 50 μL of pre‐heated elution buffer each turn, yielding 100 μL of DNA isolate. DNA quantity was evaluated using UV spectrophotometry (NanoDrop; Thermo Fischer Scientific). Samples were amplified through polymerase chain reaction (PCR) using the mitochondrial markers cytochrome *c* oxidase subunit I (COI) and 16S rRNA (16S). Each 2 μL of DNA isolate was added to a reaction mix of 21 μL Milli‐Q filtered water and each 1 μL of forward and reverse primer and aliquoted to Illustra PuReTaq Ready‐To‐Go PCR‐Beads (Avantor; VWR Int. GmbH, Darmstadt, Germany). COI was targeted using the standard barcode primers LCO1490 5′‐GGT CAA CAA ATC ATA AAG ATA TTG G‐3′ and HCO2198 5′‐TAA ACT TCA GGG TGA CCA AAA ATC A‐3′ (Folmer et al. 1994) which yielded fragments of 615 bp. Reactions were run on a thermocycler with an initial denaturation at 95°C for 5 min, followed by 38 cycles of denaturation at 95°C for 45 s, annealing at 45°C for 50 s, and extension at 72°C for 1 min. Final extension took place at 72°C for 5 min. The variable region of 16S was targeted using the forward LMY16F 5′‐GAC GAR AAG ACC CYR TCA AAC‐3′ and reverse Lu16R4 5′‐GCT GTT ATC CCT CCA GTA ACT‐3′ primers, with thermal cycling conditions applied as given in Chase et al. (1998) and Etter et al. (2011). The 16S fragments yielded 99–157 bp. Quality and quantity of amplified products were assessed by gel electrophoresis using 1.5% gels. Purification of successful PCR products was performed combining 10 μL of PCR product with 3 μL of ExoSAP‐IT PCR Product Cleanup Reagent (Thermo Fischer Scientific) and run on a thermal cycler at 37°C and 80°C for 15 min each. Purified product was sent to Macrogen Europe Inc. (Amsterdam, Netherlands) and Eurofins Genomics Germany GmbH (Ebersberg, Germany) for double stranded sequencing on ABI3730xl sequencers. DNA aliquots are stored at −80°C at the DZMB Hamburg, Germany. Forward and reverse chromatograms were edited and assembled using Geneious Prime (2023.1.2; Biomatters, Auckland, New Zealand; Kearse et al. 2012). The Basic Local Alignment Search Tool (BLAST; Altschul et al. 1990) was used to check for potential contamination and to confirm species identification.

To identify the occurrence of female and male mtDNA among our sequenced specimens, the following additional sequences from samples collected during the ENAB expedition were included: COI: 68, female mtDNA; 16S: 5, female mtDNA (HQ907901–HQ907905) and 5, male mtDNA (HQ907906–HQ907910) obtained by Boyle and Etter (2013). Among these, there are three specimens (Lu20BC2–Lu20BC4) from which three sequences were obtained per individual, being the female and male mtDNA of 16S as well as the female mtDNA of COI (Table S1; see Supplemental Methods in Appendix S1). Alignments were performed using the automated algorithm in MAFFT 7.490 (Katoh and Standley 2013) and checked by eye for quality control. Haplotype networks were inferred using the TCS algorithm (Clement et al. 2002) as implemented by PopART version 1.7 (Leigh and Bryant 2015). Kimura‐2‐Parameter distances (K2P; Kimura 1980) within and between genetic clusters were calculated using MEGA X (Kumar et al. 2018). Bayesian tree hypotheses were generated for both mitochondrial markers. The software jModelTest 2 (Darriba et al. 2012; Guindon and Gascuel 2003) was used to estimate best‐fit models of evolution by applying the Akaike Information Criterion (AIC; Sakamoto et al. 1986), resulting in GTR + I + G for COI and HKY85 for 16S. Clustering analyses of single‐gene and concatenated alignments were performed using MrBayes 3.2.1 (Huelsenbeck et al. 2001), with four parallel runs of 5 million generations, sampling every 10,000 generations. Convergence of independent runs was examined in Tracer 1.7.2 (Rambaut et al. 2018) with a burn‐in of 10%. Trees were reconstructed using Bayesian Inference (BI), assessing branch support by posterior probability (PP) with values ≥ 0.95 considered as highly supported (Felsenstein 1985; Huelsenbeck et al. 2001). The trees were rooted with the autobranch bivalve *Vesicomya galatheae* (Knudsen 1970). Delimitation of genetic clusters was complemented using the distance‐based method ASAP (Assemble Species by Automatic Partitioning; Puillandre et al. 2021), applying each of the substitution models with default settings.

#### 2.2.3 Single‐Nucleotide Polymorphism Data by 2b‐RAD

A streamlined restriction site‐associated DNA (RAD) genotyping method based on sequencing of uniform fragments produced by type IIB restriction endonucleases (2b‐RAD) was carried out using a subset of 93 samples (Table S2). Sample preparation was conducted following the protocol of Wang et al. (2012) with the restriction enzyme *BgcI*. Sequencing was performed at the Alfred Wegener Institute (Bremerhaven, Germany) on an Illumina Next‐Seq 2000 using P2 reagents and generating 50‐bp paired‐end reads. Raw reads were demultiplexed by internal barcodes and PCR duplicates removed using a custom script (https://github.com/pmartinezarbizu/2bRADpp). The data were further processed using Stacks (v 2.68; Rochette et al. 2019). Demultiplexed reads were quality filtered with a minimum quality score threshold of 30 to retain high‐confidence reads. Locus assembly and genotyping were performed using the Stacks modules. Loci were assembled for each individual (*ustacks*), with minimum stack depths (‐m) of three, five, and eight, a maximum of two mismatches (‐M) between stacks, and a limit of four loci per individual (‐N). Gapped alignment was disabled to maintain uniformity in the assembly process. Subsequently, *cstacks* was used to generate a catalog of loci based on a map file which allowed for the identification of shared loci across individuals. Using *sstacks* and *gstacks*, sequence data was aligned to this catalog, enabling the detection of matching loci and the generation of genotypic data. The *populations* module was employed with a minimum sample fraction threshold of 0.1 and one population for all samples. All downstream analyses were performed using individual SNP information (based on stacks output: populations.snps.vcf) in a custom R script (doi: 10.5061/dryad.t1g1jwtdr). Filtering thresholds were applied to remove individuals with > 75% missing genotype calls and loci with > 20% missing data. Polymorphic loci were retained by excluding singletons and non‐polymorphic sites. Data manipulation utilized the R packages vcfR (Knaus and Grünwald 2017), adegenet (Jombart 2008; Jombart and Ahmed 2011), and SNPRelate (Zheng et al. 2012). Filtered VCF files were converted to *genlight* and *genind* objects for subsequent analyses. Linkage disequilibrium (LD) pruning was performed with a threshold of *r* ^2^ = 0.2, retaining a subset of loci for downstream population structure analyses. Missing genotype data were imputed and reformatted for compatibility with the LEA (Latent Environmental Ancestry) package (Frichot and François 2015). To assess the influence of minimum stack depth on genotyping outcomes, stack depths of three, five, and eight were tested (Table S3). As expected, increasing the minimum stack depth reduced the total number of retained SNPs (2824, 2048, and 1410, respectively), while mean heterozygosity increased slightly from 0.0637 (*m* = 3) to 0.0681 (*m* = 8). All further analyses (except for conStruct) were performed with filtered SNPs from all three pipelines. Overall, we observed no major differences in downstream patterns of population structure across the three datasets. We therefore selected *m* = 5, ensuring sufficient marker density while minimizing potential genotyping errors.

Population structure was inferred using the Sparse Nonnegative Matrix Factorization (sNMF) algorithm from the LEA package. We tested values for the number of ancestral populations (*K*) ranging from 1 to 10 to identify the optimal number of populations, running 10 repetitions with 200 iterations for each value of *K*. Entropy cross‐validation was used to determine the most likely number of *K*, but did not yield a distinct minimum. Admixture bar plots were therefore generated for the most conservative probable *K* (*K* = 2) to visualize the cluster membership proportions for each individual. Population structure was additionally assessed using Bayesian clustering (*K* = 2–5, 5000 iterations, non‐spatial model) from the conStruct package (*K* = 2–5, 5000 iterations, non‐spatial model) (Bradburd 2019). Posterior distributions for the admixture proportions from four independent Markov Chain Monte Carlo (MCMC) chains were extracted and checked for label switching. Any detected label switching was manually corrected for each cluster. Subsequently, mean admixture proportions and 95% confidence intervals (CIs) were calculated. The effective sample size (ESS) for each cluster in each MCMC chain was computed using the effectiveSize function from the coda package (Plummer et al. 2006) to assess the stability of the chains. The Gelman–Rubin diagnostic was performed to evaluate convergence across chains, with the potential scale reduction factor (PSRF) calculated and visualized.

Pairwise Nei's genetic distances (Nei 1972) were calculated at the individual level based on allele frequencies derived from multilocus genotypes within and between basins using the StAMPP package (Pembleton et al. 2013). A discriminant analysis of principal component (DAPC) was performed using the three main hierarchical clusters (based on Nei distances and ward.D) as the discriminant factor. The number of included principal components was based on a‐score optimization (10 PCs and 2 discriminant functions). In addition, Nei's genetic distances were calculated pairwise between populations, based on population‐level allele frequencies. The fixation index (*F*_ST_) was calculated based on Nei and Chesser's (1983) corrected genetic differentiation statistic, which accounts for genetic differentiation among populations by incorporating heterozygosity and adjusting for sample size biases, using the R package FinePop2 (Nakamichi et al. 2020). Nei distances and *F*_ST_ were visualized as a heatmap using the pheatmap package (Kolde 2019). To better visualize connectivity between basins, we mapped *F*_ST_ values alongside admixture proportions inferred from sNMF.

#### 2.2.4 Proteomic Fingerprinting

Matrix‐assisted laser desorption/ionization time‐of‐flight mass spectrometry (MALDI‐TOF MS) was performed to obtain proteomic mass spectra, following the protocol given in Rossel et al. (2024). For each specimen, three mass spectra were measured. Raw spectral data from MALDI‐TOF MS analyses were processed and analyzed using R (R Core Team 2024), utilizing the following packages: MALDIquant, MALDIquantForeign, adegenet, randomForest, vegan, reshape2, pheatmap, ggplot2, splus2R, RColorBrewer, plyr, and dplyr (Burns 2020; Gibb 2022; Gibb and Strimmer 2012; Jombart 2008; Kolde 2019; Liaw and Wiener 2002; Neuwirth 2022; Oksanen et al. 2019; Wickham 2023b, 2020, 2023a; Wickham et al. 2022). Spectral data were imported using the importBrukerFlex() function, with empty spectra removed. Spectra were trimmed to a mass range of 2000–20,000 m/z, and intensity values were square‐root transformed to stabilize variance. Intensity smoothing was performed using the Savitzky–Golay algorithm (Savitzky and Golay 1964; window size = 10). Baseline correction was carried out using the SNIP algorithm (Ryan et al. 1988) with 15 iterations, followed by total ion current (TIC) normalization to adjust for differences in signal intensity across spectra. Technical replicates were averaged based on sample identifiers extracted from metadata. Averaging was performed using the mean intensity values of replicate spectra. Peaks were detected with a signal‐to‐noise ratio (SNR) threshold of 8, using a moving average (MAD) approach. Peak binning was conducted iteratively using a strict tolerance of 0.002 to group peaks with similar m/z values. The binning process was repeated until the number of binned peaks remained constant. To remove noise, a lower detection threshold was defined based on the relationship between peak intensity and mean spectral noise. Peaks below 1.75 times the average noise level were set to zero. Spectral intensities were further normalized using the Hellinger transformation (Legendre and Gallagher 2001), and a final intensity matrix was created by averaging peak intensities across biological replicates. Metadata for each sample, including mitotype, region, and group, were integrated into the matrix from an external reference table. DAPC was performed to identify population structure using successive k‐means clustering based on all 120 PCs. The optimal cluster number was determined based on Bayesian Information Criterion values. A Random Forest (RF) model (Breiman 2001) was employed to classify samples into regional groups based on spectral data. Samples were grouped into two broad regions, and RF classification was conducted using 10,000 trees and 22 variables per split. Hierarchical clustering based on Euclidean distances was performed on normalized peak intensities. Feature importance was assessed using Mean Decrease Accuracy, and the top 13 peaks (with values > 0.0006) were selected for presentation as a heatmap using the pheatmap package.

## 3 Results

### 3.1 Mitochondrial Markers

Mitochondrial markers were successfully amplified for 135 specimens of *L. ultima* , yielding 235 novel sequences for the two markers COI (128 sequences; 615 bp) and 16S (107 sequences; 99–157 bp). In total, 100 specimens were successfully sequenced for both markers. Sequence data are accessible in the Barcode of Life Data System (BOLD v4; Ratnasingham et al. 2024) via 10.5883/DS‐LEDUL. GenBank accession numbers are listed in Table S2.

**FIGURE 3:**
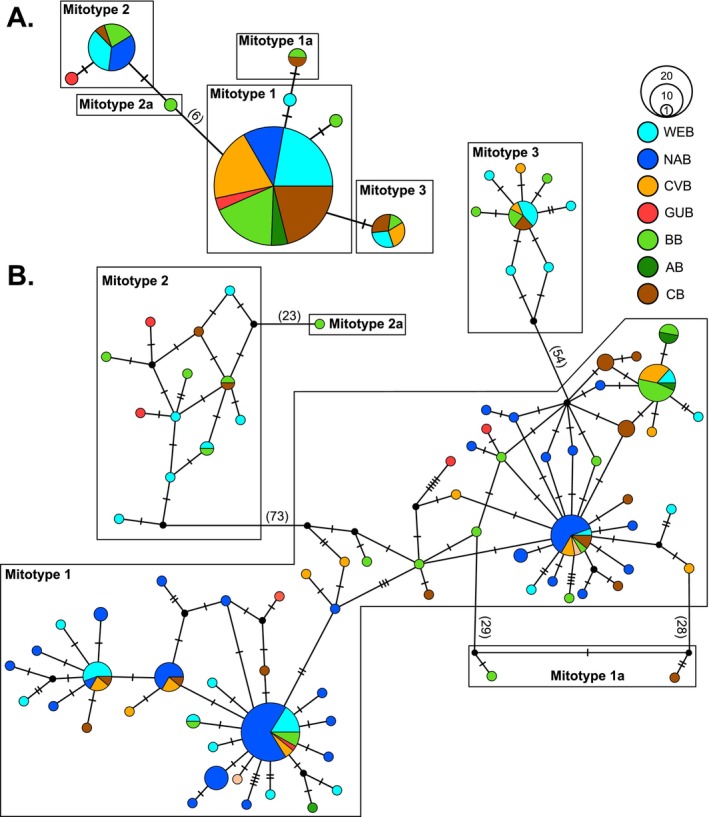
TCS haplotype network analyses of *L. ultima* resulting in each of the five groups of mitochondrial haplotypes (mitotypes) for the genetic markers (A) 16S and (B) COI. Colors correspond to the respective Atlantic basins. Hash marks refer to the number of mutational steps between specimens. Filled black circles correspond to missing haplotypes.

The TCS haplotype network analyses for both mitochondrial markers (Figure 3) each resulted in five groups of mitochondrial haplotypes, hereafter referred to as mitotypes (MTs). Identification of MTs was based on the number of nucleotide substitutions (23–73) revealed by COI between MT 1 and MTs 1a, 2, 2a, and 3, and were, respectively, transferred to the less divergent network of 16S (Figure 3A). For both markers, samples were consistently allocated to respective mitotypes but were distributed across clusters and basins without clear geographic or genetic correspondence (Figure 3A,B). A total of 94 specimens spanning all Atlantic basins clustered as MT 1, including 94 sequences for COI and 88 sequences for 16S. Additional female mtDNA sequences from the NAB (COI: 68, 16S: 5, Table S1) congruently clustered with MT 1. Fourteen specimens were assigned to MT 2, including 14 sequences for COI and eight sequences for 16S, covering all basins but the CVB and the AB. In addition, five male mtDNA 16S sequences from the NAB (Table S1) clustered with MT 2. Mitotype 3 yielded a total of 16 specimens, including 16 COI and eight 16S sequences, covering the WEB, CVB, BB, and the CB. Furthermore, for both markers, one specimen from the BB and the CB was identified as MT 1a, and a single specimen from the BB was identified as MT 2a. The ASAP analyses of COI (Figure S1) and 16S (Figure S2) delimited partitions congruent with the haplotype network analyses but scored MT 1a and MT 2a either as separate clades or grouped with MT 1 and MT 2, respectively. K2P distances within mitotypes yielded a maximum of 0.05 for both markers (Table 2). Distances between mitotypes were found lowest for the comparisons of MT 1 and MT 1a (COI: 0.08–0.11, 16S: 0.02–0.08) and MT 2 and MT 2a (COI: 0.06–0.07, 16S: 0.02–0.05). Except for the distance between MT 1 and MT 3 for 16S (0.05–0.10), distances were found to range between 0.16–0.30 for COI and 0.10–0.25 for 16S.

**TABLE 2:** K2P distances within (in italics) and between mitotypes (MT) identified by the mitochondrial markers COI and 16S.

	COI					16S				
	MT 1	MT 1a	MT 2	MT 2a	MT 3	MT 1	MT 1a	MT 2	MT 2a	MT 3
MT 1	*0.00–0.04*					*0.00–0.05*				
MT 1a	0.08–0.11	*0.00–0.01*				0.02–0.08	*0.00*			
MT 2	0.23–0.26	0.25–0.26	*0.00–0.02*			0.18–0.25	0.22–0.25	*0.00–0.02*		
MT 2a	0.24–0.27	0.22–0.24	0.06–0.07	*0.00*		0.15–0.19	0.19–0.20	0.02–0.05	*0.00*	
MT 3	0.16–0.19	0.17–0.18	0.26–0.28	0.29–0.30	*0.00–0.01*	0.05–0.10	0.10–0.12	0.18–0.25	0.15–0.19	*0.00–0.02*

### 3.2 2b‐RAD Analyses

**FIGURE 4:**
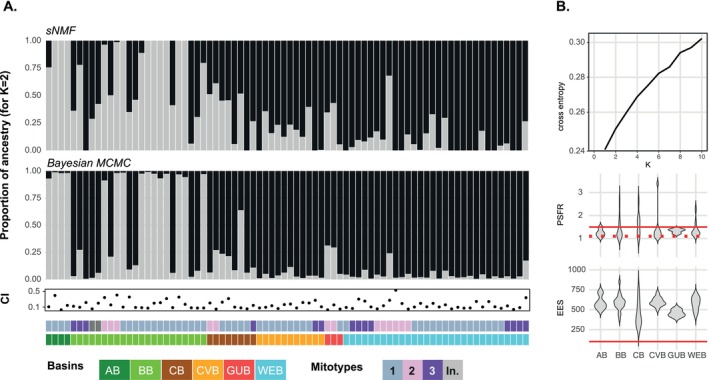
Patterns of population structure for *L. ultima* based on SNP data obtained by 2b‐RAD sequencing. (A) Proportions of ancestry for *K* = 2 using the Sparse Nonnegative Matrix Factorization (sNMF) algorithm and Bayesian clustering, with posterior distributions for admixture proportions from four independent Markov Chain Monte Carlo (MCMC) chains. For each cluster, mean admixture proportions and 95% confidence intervals (CIs) were calculated. Colored bars correspond to mitotypes (top) and six Atlantic basins (bottom). (B) Cross‐entropy criterion, potential scale reduction factor (PSRF) calculations, and effective sample sizes (ESS) across *K* values from 1 to 10.

For the applied stack depth *m* = 5, mean coverage per individual across 14,220 loci was 42X (Table S3). These values are in line with expected coverage for 2bRAD datasets (Wang et al. 2012). After quality filtering and SNP calling, a total of 2048 polymorphic loci were retained across 78 individuals. Samples were distributed across six basins, with the following sample sizes per region: WEB (*n* = 30), GUB (*n* = 3), CVB (*n* = 11), BB (*n* = 22), AB (*n* = 4), and CB (*n* = 8). No individuals from the NAB met the filtering thresholds and were thus excluded from further analysis. The sNMF analysis did not identify a distinct minimum in the cross‐entropy criterion across *K* values from 1 to 10 (Figure 4A), indicating the absence of strong population subdivision. Both sNMF and Bayesian clustering (conStruct) yielded largely congruent ancestry coefficients (Figure 4B) for a scenario of *K* = 2. After evaluation of a wider range of *K* (Figure S3), we selected this scenario as the most parsimonious model based on comparatively lower entropy but also on biological relevance. This rather conservative approach acknowledges that the observed consistency in ancestry patterns may be shaped by large‐scale oceanographic features. Specifically, the observed clustering aligns with major deep‐sea current systems that may shape gene flow among abyssal basins. The NADW predominantly flows southward along the western Atlantic margin, facilitating potential connectivity among the WEB, GUB, and CVB, where individuals predominantly cluster with K1 (black). In contrast, connectivity of South Atlantic basins, specifically the AB, may be more strongly influenced by the AABW. Individuals from AB were predominantly assigned to the second cluster K2 (gray), forming a distinct genetic group. Intermediate proportions of admixture were found in the BB and CB regions. MCMC convergence was confirmed for all conStruct runs with PSRF values < 1.1 and ESS > 250 for all *K* values (Figure 4C–E). Although CIs were high for a small subset of individuals, the data suggest adequate convergence and stability of the admixture estimates. To explore whether mitochondrial haplotype distributions mirrored genomic population structure, mitotypes were overlaid onto the admixture results (Figure 4A). Mitotypes were distributed across clusters and basins without clear geographic or genetic correspondence. Two mitotypes (1a and 2a), represented by single individuals, were considered indeterminate and excluded from population‐level interpretation.

**FIGURE 5:**
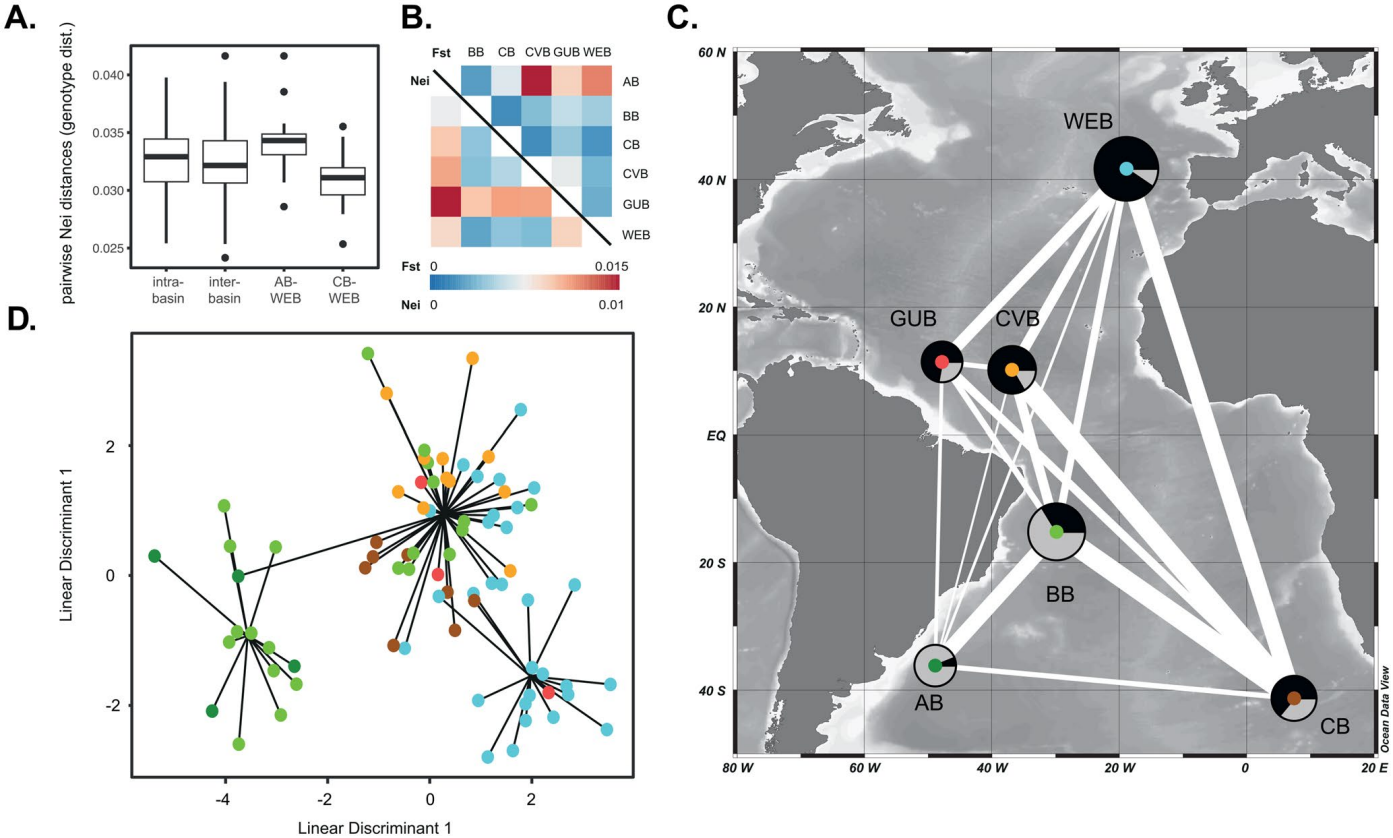
Genetic distance based on SNP data calculated from LD‐pruned, polymorphic loci. (A) Calculated pairwise Nei's genetic distances among individuals for intra‐ and inter‐basin comparisons, including comparisons between the geographically most distant basins. (B) Heatmap of *F*_ST_ values (upper) and Nei's genetic distance (lower), using a color gradient between red for smallest and blue for largest values. (C) Illustrated view of proportions of ancestry for *K* = 2, inferred from the Sparse Nonnegative Matrix Factorization (sNMF) algorithm averaged for each basin. Circle size indicates sample size; line width is inversely proportional to genetic differentiation (*F*_ST_) between basins. Map generated with Ocean Data View (Schlitzer 2022). (D) Inferred clusters by DAPC from hierarchical Ward clustering on pairwise Nei's genetic distances. Colors represent respective Atlantic basins.

Pairwise Nei's genetic distances among individuals were calculated separately for within‐ and between‐basin comparisons, showing substantial overlap (Figure 5A). However, certain between‐basin comparisons showed elevated values, most notably between AB and WEB, which exhibited some of the highest pairwise genetic distances among all basin pairs. *F*_ST_ values calculated from LD‐pruned, polymorphic loci ranged from 0.002 to 0.018, indicating overall low levels of genetic differentiation across regions. The highest values were observed between AB and CVB (*F*_ST_ = 0.018), followed by AB–WEB and AB–GUB comparisons. In contrast, minimal differentiation was detected between WEB, GUB, and CVB. These patterns are visualized as an *F*_ST_ heatmap (Figure 5B), highlighting subtle but consistent genetic structure among basins. Nei's genetic distances between populations revealed similar patterns of differentiation (Figure 5B), except for elevated distances involving GUB. These values should be interpreted with caution, as unbalanced sample sizes may have inflated estimates of genetic divergence (Kitada et al. 2021). The mapped *F*_ST_ values alongside admixture proportions inferred from sNMF, averaged for each basin, illustrate the extent of connectivity between populations by geographic distance (Figure 5C), with line widths inversely proportional to genetic differentiation (*F*_ST_). The DAPC, using inferred clusters from hierarchical Ward clustering on pairwise Nei's genetic distances, supported the separation of AB from all other basins (Figure 5D). The first discriminant function accounted for most of the inter‐basin variance and separated AB individuals from those in the WEB, GUB, and CVB. The BB and CB samples occupied an intermediate position along this axis.

### 3.3 Proteomic Fingerprinting

**FIGURE 6:**
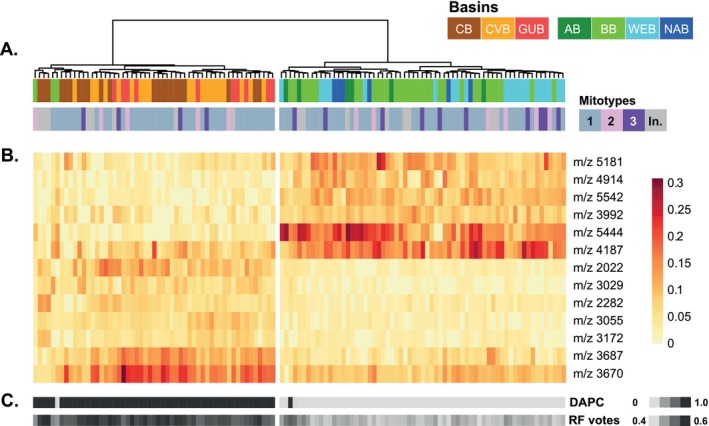
Proteome fingerprinting results from specimens of *L. ultima* across seven Atlantic basins. (A) Clustering results based on the relative proteomic composition across samples. Colored bars correspond to basins (top) and mitotypes (bottom). (B) Heatmap of relative intensities of mass peaks for differentiation at the population level, identified using the RF classifier. (C) Probability of assignment for the RF model and the DAPC analysis, revealing a solution of two k‐means clusters.

In total, 120 specimens were used in the MALDI‐TOF MS analysis. Their clustering based on relative proteomic composition resulted in two main groups (Figure 6A). One group included all specimens sampled in the central Atlantic (GUB and CVB) and the south‐east Atlantic (CB), as well as an additional three samples from the south‐west Atlantic (BB). The second group clustered all specimens from the north Atlantic (WEB and NAB) and the south‐west Atlantic (AB), as well as all remaining samples from the BB. A RF model based on these two groups (out of box error: 0.03) revealed mass peaks that are considered important for the differentiation between groups. While all peaks were found in specimens from both groups, between‐group peak intensities differed distinctly (Figure 6B). Within the RF model, every specimen received a probability of assignment to the CB_CVB_GUB class, ranging from 0.4 to 0.6 (Figure 6C). DAPC analysis revealed a solution of two k‐means clusters and was able to discriminate all but two specimens into the same groups as the hierarchical clustering. Mitotypes delineated by the mtDNA analyses were included for reference and resulted in a scattered distribution across basins and groups. Clustering results were checked against shell morphometric measurements, including length, width, and levels of ventral edge thickening (data not shown). No correlation was found.

## 4 Discussion

Low genetic differentiation across abyssal plains has been hypothesized to result from an interplay of reduced habitat heterogeneity, slow evolutionary rates, few topographic barriers, and extensive gene flow between distant populations (Etter et al. 2005; Rex and Etter 2010; Jennings et al. 2013). Yet, our understanding of population structure and connectivity in these understudied habitats remains scarce. Subtle population structures that may exist in such environments can remain undetected when relying solely on mitochondrial markers like 16S or COI, which often lack the resolution to capture fine‐scale genetic patterns (Andrews et al. 2016; Hurst and Jiggins 2005; Reitzel et al. 2013). To overcome these limitations, we combined mitochondrial markers with genome‐wide SNP data and proteomic fingerprinting to assess whether subtle population structure and potential ecological subdivision exist in the abyssal bivalve *L. ultima* across major Atlantic basins.

### 4.1 Phylogeographic Patterns and Population Connectivity

The analyses of SNP data detected subtle genetic structure, despite uniformly low absolute *F*_ST_ values across basins (0.002–0.018). Comparably low *F*_ST_ values have been reported in other deep‐sea mussel species (Xu et al. 2017; Yao et al. 2022). Although numerically small, this approximately tenfold variation in *F*_ST_ estimates indicates biologically meaningful structure when interpreted in the context of overall low heterozygosity in the data, as well as high‐dispersal potential and large effective population sizes (*N*_e_). This emphasizes the necessity of applying genome‐wide markers to resolve fine‐scale population structure, as has been demonstrated for different deep‐sea organisms (Diaz‐Recio Lorenzo et al. 2024; Galaska et al. 2017; Pante et al. 2015; Takata et al. 2021; Xu et al. 2018). Generally, population sizes of *L. ultima* across the abyssal Atlantic are huge (Allen 2008; Allen and Hannah 1989), with an estimated *N*_e_ of more than 10 million individuals for the NAB alone (Etter et al. 2011). It is likely that the high number of reproducing individuals weakens the effects of genetic drift and contributes to the maintenance of the overall similar genetic architecture seen in our data, where a general concordance between *F*_ST_ patterns and the pairwise Nei's genetic distance between populations suggests that historical divergence and current gene flow contribute at a similar scale (Marko and Hart 2012).

The extensive gene flow between distant populations of *L. ultima* might seem counterintuitive at first (Etter et al. 2011), especially because the overall slow abyssal currents would rather be suggestive of limited dispersal distances (Stow et al. 2019; Zenk 2008). This perspective aligns with the long‐standing paradigm that pelagic lecithotrophs are altogether less dispersive than planktotrophs (Calow 1983; Jablonski and Lutz 1983; O'Connor et al. 2007). Especially for deep‐sea taxa, however, it has repeatedly been shown that lecithotrophic development itself does not constrain dispersal (Levin 2006; Weersing and Toonen 2009; Young et al. 1997). For example, in echinoderm lecithotrophs, not only was the duration of the free‐swimming phase found to be longer when coupled to lowered temperatures (Mercier et al. 2013), but also the swimming‐speed capacity increased when compared to planktotrophic larvae of comparable size (Montgomery et al. 2019). In *L. ultima* , the development of few, large‐sized eggs per female is balanced by high population densities (Allen and Hannah 1989; Scheltema 1972). Since more energy per egg is provided compared to planktotrophs, and temperatures at abyssal depths are constantly low, lecithotrophic larvae likely have the ability to delay developmental rates and increase their dispersal rates (Scheltema 1972; Young 2003; Scheltema and Williams 2009; Jennings et al. 2013). Given that this numerically abundant species has rarely been sampled above 3000 m, it seems plausible that populations of *L. ultima* continuously produce vital numbers of recruits, sufficient to maintain self‐sustained populations, replenish adjacent populations, and maintain gene flow by passive transport processes of pelagic lecithotrophic larvae across the continuous abyssal plains. We postulate that deep‐ocean circulation dynamics and the use of geographically intermediate soft‐bottom habitats are crucial for the maintenance of genetic connectivity in this tiny pan‐Atlantic bivalve. Furthermore, periodic strong bottom flows in northern and southern basins, with the potential to sweep larvae and juveniles together with surficial organic matter across the seabed (Gardner et al. 2017; Hollister and McCave 1984; Thistle et al. 1991; Zenk 2008), may be an additional driver for the maintenance of connectivity between populations in the deep sea (Aller 1989; Gheerardyn and Veit‐Köhler 2009; Harris 2014; Meißner et al. 2023).

Our SNP analyses recovered highest *F*_ST_ values between AB–WEB, AB–GUB, and AB–CVB. Together with a concordant signal in Nei values for AB, this suggests a genuine pattern of differentiation. Elevated Nei distances at low *F*_ST_ values in the GUB are more likely attributable to sampling artifacts caused by unbalanced datasets than to actual genetic divergence. We observed low genetic divergence between the geographically distant basins WEB and CB; however, markedly higher levels of genetic differentiation between the similarly distant basins WEB and AB (Figure 5A–C). This implies that isolation by geographic distance is not acting as a key driver and evokes an important distinction between the geographic and the effective distance between basins (Dambach et al. 2016; McClain and Hardy 2010; Xuereb et al. 2018). Whereas distance is often interpreted by means of a two‐dimensional scale, the direction and strength of bottom currents in the three‐dimensional marine realm have a direct effect on the potential for dispersal and thus gene flow between populations (Lecroq et al. 2009; Menzel et al. 2011; Miller and Gunasekera 2017). This implies that the deep‐water circulation patterns of AABW and NADW provide direct but non‐uniform links between populations in the Atlantic. While the propagation of NADW connects the north and central Atlantic region, as well as the south‐west African margin, the AABW mainly links the west‐Atlantic margins, including parts of the north‐eastern corridors of the MAR facilitated by deep abyssal channels (Ferreira and Kerr 2017; Garzoli and Matano 2011; Morozov et al. 2010). As for the low genetic divergence between WEB and CB and the proportions of ancestry revealed by our analysis, we propose the genetic admixture to be driven by the north‐east directional flow of NADW. Upon its crossing of the MAR through extensive fracture zones in the central Atlantic (German et al. 2011; Morozov et al. 2010), most of NADW is retained in the Angola Basin through advective currents caused by the Walvis Ridge (Bartels 2008; Shannon and Chapman 1991). As demonstrated for polychaetes (Fiege et al. 2010), harpacticoid copepods (Menzel et al. 2011) and isopods (Brix et al. 2011, 2015; Brökeland 2010), the Walvis Ridge does not appear as an absolute barrier for connectivity, allowing a fraction of bottom water to enter the CB. While similar patterns of genetic connectivity across ocean basins have been shown for brooding isopods (Bober et al. 2018; Brix et al. 2011, 2015), indicating bottom‐water masses to function as vectors for population connectivity, the 16S data analysis by Etter et al. (2011) revealed modest genetic divergence between populations of *L. ultima* from eastern and western basins of the North Atlantic, suggesting the MAR to function as a topographic barrier to gene flow. Since we do not see such distinctive barriers to gene flow in our genome‐wide analysis of population structure, we interpret these patterns as basin‐based divisions with possible small‐scale effects of the MAR instead.

The concept of effective distance can be developed further by integrating the potential for asymmetric gene flow, where migration between populations follows a unidirectional fashion mediated by current flows and strong advection processes (Snead et al. 2023; Stow et al. 2019; Xuereb et al. 2018). For the south‐east Atlantic basins AB and BB, our analysis results of sNMF and Bayesian MCMC congruently found an overall mixed proportion of ancestry for K1 and K2 in the BB, and a predominance of K2 in the AB (Figure 4A). These findings are indicative of asymmetric gene flow mediated by the intensity and directional flows of AABW and NADW, possibly evoking a unidirectional migration from the AB population into the BB population, and the result of fine‐scale genetic structure observed. While a large fraction of AABW passes the Rio Grande Rise through the Vema Gap into the BB (Morozov et al. 2010, 2023), minimizing the effective distance, the southward flow of NADW into the AB is largely restrained by retentive hydrodynamic forces (Alberoni et al. 2020; Perez et al. 2020), increasing the effective distance between these basins. While the predominance of K2 in the AB and indications of asymmetric gene flow could suggest this basin to serve as a potential site for ongoing speciation processes, and a fraction of individuals from the BB showed a proportion of ~90% of either K1 or K2, implying patterns of assortative reproduction, we advise careful interpretation of these findings. Given that the sNMF clustering revealed generally higher mixing proportions in the BB compared to the Bayesian MCMC, and we generally found low genetic differentiation across basins, we already discussed population structure at a fine scale where the risk for overinterpretation is almost inevitable.

### 4.2 Proteomic Fingerprinting

Proteomic analyses revealed two distinct groups: one comprising the central and south‐east Atlantic (GUB, CVB, CB) and the other spanning the north‐ and south‐west Atlantic (WEB, NAB, BB, AB). This division contrasts with the genetic structure inferred from SNP data but aligns with the scattered distribution of mitotypes across basins and groups. Given that previous proteomic studies have been successfully applied for species identification (Paulus et al. 2022; Peters et al. 2023; Renz et al. 2021), differentiation of cryptic lineages (Kaiser et al. 2018), and distinguishing reproductive stages at the species level (Rossel et al. 2023), our findings show that the genetic divergence between mitochondrial haplotypes does not correspond to proteomic differentiation and support the presence of a single species. A test for correlation between clustering results and the distribution of shell size (width, length) and the extent of ventral edge thickening (see Figure 2) did not reveal any congruence and is further supportive of a single‐species hypothesis (personal observation, data not shown). We simply tested this to implement the little we know about the ecology of *L. ultima* , producing a few large eggs when exceeding a length of 2.4 mm, upon which the edge thickening initiates (Allen and Hannah 1989; Tyler et al. 1992). As the thickening increases the shell volume and space for eggs, it could have served as a proxy for reproductive activity and sexual maturation.

We suggest that the observed proteomic variation in *L. ultima* reflects environmentally driven shifts in protein expression at the intraspecific level, potentially caused by differences in particulate organic carbon (POC) flux to the abyssal seafloor. Specifically, we hypothesize that the first group (GUB, CVB, CB) experiences lower annual POC flux than the second group (WEB, NAB, BB, AB). Existing data support this hypothesis to some extent, albeit with significant gaps in our understanding. The CB is considered an oligotrophic environment, with estimated annual POC fluxes of less than 1 g C_org_ m^−2^ y^−1^ (Schmiedl et al. 1997; Watling et al. 2013). Similarly, nutrient availability in the central Atlantic near the CVB (18° N 21° W) has been described as low (Antia et al. 2001). However, seasonal blooms in the eastern equatorial Atlantic have been reported to enhance POC flux in this region (Lutz et al. 2007; Pérez et al. 2005). The north‐ (WEB, NAB) and south‐west Atlantic (BB, AB) generally exhibit moderate to high POC fluxes (2–6 g C_org_ m^−2^ y^−1^; Watling et al. 2013; Lampitt et al. 2023), suggesting a relatively productive abyssal environment that potentially supports higher metabolic activity and proteomic signatures distinct from the central and south‐east Atlantic. While these observations suggest a link between POC flux and proteomic differentiation, it remains speculative due to the limited availability of direct measurements of POC flux and environmental conditions across these basins. Additional long‐term, spatially resolved biogeochemical studies are necessary to validate this potential correlation. An integration of epigenetic analyses could further help to determine whether the observed proteomic shifts are driven by transient physiological responses or stable adaptive mechanisms.

### 4.3 Mitochondrial Inheritance Patterns

The analysis of the COI marker identified five mitotypes without clear geographic or genetic correspondence. We interpret these patterns to result from sex‐specific heteroplasmic mtDNA, which has repeatedly been shown to challenge assessments of genetic lineages and evolutionary inference across marine taxa (Chow et al. 2021; Shigenobu et al. 2005; Vollmer et al. 2011) and bivalves specifically (Capt et al. 2020; Ghiselli et al. 2021; Passamonti and Ghiselli 2009; Robicheau et al. 2017; Zouros and Rodakis 2019). We therefore propose that robust assessments of regional genetic structure based on mitochondrial data alone are only possible between specimens of the same mitotype, rather than across the highly divergent mitochondrial lineages. For the 16S marker in particular, only a single main haplotype was detected within each mitotype, highlighting the limited resolution of 16S for assessing regional differentiation in this species (Etter et al. 2005, 2011) and other metazoan taxa (Cho and Shank 2010; Miller et al. 2010; Neal et al. 2018; Thornhill et al. 2008).

Besides the presence of heteroplasmic mtDNA, many bivalves exhibit a peculiar mitochondrial inheritance system called doubly uniparental inheritance (DUI), which maintains two sex‐specific mtDNA types within a single individual (for details, see Breton et al. 2007; Gusman et al. 2016; Guerra et al. 2017; Ghiselli et al. 2021; Smith et al. 2023). Under DUI, females transmit their mtDNA (F‐type) to both sexes, while males pass theirs (M‐type) to sons only (Passamonti and Ghiselli 2009; Smith et al. 2023). Thus, males are heteroplasmic for their mtDNA, with the M‐type typically in gonads and the F‐type in somatic tissue, although this ratio varies by species and tissue type (Garrido‐Ramos et al. 1998; Machordom et al. 2015; Obata et al. 2006; Passamonti and Scali 2001; Sano et al. 2007). Overall, intraspecific divergence between F and M mtDNA may vary from 10% to over 50% (Breton et al. 2007; Capt et al. 2020; Passamonti and Ghiselli 2009; Robicheau et al. 2017; Zouros 2013), for example in the species *Mytilus edulis* , *M. trossulus* , and *M. californianus* (10%–20%; Zouros 2013), *M. modiolus* (37%–40%; Robicheau et al. 2017), and the freshwater mussel *Quadrula quadrula* (52%; Doucet‐Beaupré et al. 2010). The prevalence of DUI further complicates the transmission of female and male mtDNA. Mitochondrial role‐reversal, for instance, can lead to genome masculinization, where female mtDNA displaces the M‐type and establishes itself as a new male lineage (for details, see Hoeh et al. 1997; Theologidis et al. 2008; Stewart et al. 2009; Sańko and Burzyński 2014; Gusman et al. 2016). Because such events can reset F‐ and M‐type divergence to zero and enable new divergence patterns, the F‐ and M‐types within males can show high divergence or be nearly identical (Hoeh et al. 1997; Quesada et al. 1999; Stewart et al. 2009; Theologidis et al. 2008; Zouros 2013). The rapid evolution of male mtDNA may reduce sequence similarity to standard mitochondrial primers, rendering it undetectable by PCR. In contrast, the slower‐evolving F‐type retains similarity to universal primers, increasing its amplification likelihood (Hoeh et al. 2002).

Collectively, DUI has been attested in at least seven bivalve families (Doucet‐Beaupré et al. 2010; Gusman et al. 2016; Theologidis et al. 2008; Walker et al. 2006) and has been suggested as the likely source for previously evidenced mitochondrial heteroplasmy in *L. ultima* (Boyle and Etter 2013). Therein, mitochondrial heteroplasmy was attested by the use of specifically targeted mitochondrial fragments and cloning. From about half of their specimens, Boyle and Etter (2013) amplified both F‐ and M‐types of mtDNA, showing up to 27% divergence, whereas the remaining individuals yielded only female mtDNA. This degree of divergence falls within the range observed in bivalves with DUI (e.g., Gusman et al. 2016; Robicheau et al. 2017), as well as the genetic intraspecific divergence of up to 30% observed in our data (Table 2). However, experimental validation of DUI in deep‐sea protobranchs is presently impractical, as they are difficult to sustain under laboratory conditions and are likely to have long generation times (Boyle and Etter 2013; Turekian et al. 1975; Zardus 2002). Although likely that DUI is the source for mitochondrial heteroplasmy in *L. ultima* , the current state of knowledge on the genetic complexity in this species does not yet allow for final conclusions on the presence of DUI. Yet, we highlight this genetic complexity at the intraspecific level, as it can lead to overestimations of species diversity due to misinterpreted genetic distances in *L. ultima* , as seen in other taxa (Chow et al. 2021; Martínez et al. 2023; Wai Ho and Hanafiah 2024). When examined independently from SNP and proteomic data, the large genetic distances may suggest multiple lineages or cryptic species *of L. ultima*. However, this is misleading for several reasons. Our data indicate that the observed genetic divergence of up to 30% is owed to heteroplasmic mtDNA, since all sequences from the NAB, pre‐defined to female and male mtDNA, clustered with MT 1 and MT 2, respectively (Table S1). Mitotype 1 likely reflects female mtDNA from either homoplasmic females or heteroplasmic males with a dominant F‐type. Thus, MT 1 likely includes specimens of both sexes, whereof the female mtDNA was sequenced. A fraction of the M‐type may have gone undetected by universal primers. However, this is unlikely as MT 2 clustered with pre‐sequenced male mtDNA from the NAB. We suggest that the predominant M‐type of specimens in MT 2 has likely been sequenced from mature individuals with higher fractions of gonadal tissue. Morphometric shell measurements of the 14 specimens from MT 2 revealed a shell length of 2.6–3.0 mm (data not shown), consistent with the minimum shell length of 2.4 mm for *L. ultima* to initiate gonadal development (Allen and Hannah 1989). The presence of MT 3 and the mitotypes 1a and 2a complicates interpretation. Since masculinized mtDNA could sequentially evolve into new M‐types (Hoeh et al. 2002; Sańko and Burzyński 2014; Stewart et al. 2009), we propose these mitotypes to represent ongoing role‐reversal or transitional states of the established F‐ and M‐types. Specifically targeted sequencing and cloning aligned with the protocols in Boyle and Etter (2013) on sexed specimens are encouraged to test this hypothesis in future applications.

### 4.4 Conclusions and Implications for Conservation

Facilitated by the decadal sampling hubs of the aforementioned international programs, the pan‐Atlantic collection of *L. ultima* has enabled a multidisciplinary study of population connectivity at abyssal depths. While mitochondrial data suggested inflated genetic divergence due to heteroplasmic inheritance and potential DUI mechanisms, our nuclear SNP and proteomic analyses revealed overall low genetic divergence with support of a single species. Subtle yet significant population structure was identified, indicating two genetically connected but distinguishable source populations in the northern/central and southern Atlantic, with admixture zones in the BB and CB. This genetic structuring did not correlate with mitotype distribution, but rather reflected patterns consistent with gene flow driven by abyssal circulation and asymmetric gene flow mediated by the northward trajectory AABW. Furthermore, proteomic differentiation pointed to ecological divergence in protein expression, potentially linked to regional differences in POC flux. Our findings highlight the limitations of mitochondrial markers in the presence of complex inheritance systems and underscore the value of integrating nuclear genomic and proteomic tools to decipher population connectivity in abyssal species. In addition to this population genetic approach, these samples have facilitated the assessment of *L. ultima* as Least Concern on the IUCN Red List (de Wilt 2024), offering important information for the growing conservation efforts in deep‐sea ecosystems (Sigwart et al. 2019, 2023). With prospective Atlantic deep‐sea mining activities (Amorim et al. 2024; Dunn et al. 2018; Hein et al. 2013) and environmental impacts of climate change (Ramirez‐Llodra et al. 2010; Samuelsen et al. 2022) posing imminent threats to deep‐sea biodiversity, this study highlights the critical need for sustained long‐term international monitoring to inform future conservation measures for deep‐ocean ecosystems, where our understanding of evolutionary and ecological processes still remains in its infancy.

## Author Contributions

**Jenny Neuhaus:** conceptualization (equal), data curation (lead), formal analysis (lead), investigation (lead), methodology (equal), project administration (lead), visualization (equal), writing – original draft (lead), writing – review and editing (lead). **Mark E. de Wilt:** data curation (equal), formal analysis (equal), investigation (equal), project administration (equal), software (supporting), validation (supporting), writing – review and editing (supporting). **Sven Rossel:** data curation (supporting), formal analysis (supporting), methodology (supporting), writing – review and editing (supporting). **Saskia Brix:** conceptualization (lead), funding acquisition (lead), project administration (supporting), resources (supporting), supervision (lead), writing – review and editing (supporting). **Ron J. Etter:** data curation (supporting), validation (supporting), writing – review and editing (supporting). **Robert M. Jennings:** data curation (supporting), resources (supporting), writing – review and editing (supporting). **Katrin Linse:** conceptualization (lead), resources (supporting), supervision (supporting), writing – review and editing (supporting). **Pedro Martínez Arbizu:** formal analysis (supporting), funding acquisition (lead), methodology (supporting), software (supporting), supervision (lead), writing – review and editing (supporting). **Martin Schwentner:** formal analysis (supporting), software (supporting), validation (supporting), writing – review and editing (supporting). **Janna Peters:** formal analysis (supporting), methodology (supporting), software (equal), validation (equal), visualization (supporting), writing – original draft (supporting), writing – review and editing (supporting).

## Disclosure

Benefit‐Sharing Statement: A research collaboration was developed with international scientists providing genetic samples. All collaborators are included as co‐authors. Benefits from this research accrue from the sharing of our data and results on public databases as described above.

## Conflicts of Interest

The authors declare no conflicts of interest.

## Supporting information


**Figure S1:** ece371903‐sup‐0001‐FiguresS1.zip.

**Table S1:** ece371903‐sup‐0002‐TableS1.xlsx.

**Table S2:** ece371903‐sup‐0002‐TableS2.xlsx.

**Table S3:** ece371903‐sup‐0004‐TableS3.xlsx.

**Appendix S1:** ece371903‐sup‐0005‐AppendixS1.docx.

## Data Availability

Novel mitochondrial DNA sequences are deposited to the NCBI Nucleotide Database under accessions PQ179865–PQ179990 (COI) and PV131957–PV132063 (16S). Metadata, images, and raw sequence data are deposited to the Barcode Of Life Database (10.5883/DS‐LEDUL). Raw sequence reads from 2b‐RAD are deposited in the SRA (BioProject PRJNA1245090) under accessions SAMN47739826–SAMN47739903. Individual genotype data, proteomic data, and R scripts are available on DataDryad (10.5061/dryad.t1g1jwtdr).
