# Supplementary material for: High Connectivity at Abyssal Depths: Genomic and Proteomic Insights Into Population Structure of the Pan‐Atlantic Deep‐Sea Bivalve Ledella ultima (E. A. Smith, 1885)
Source: Ecol Evol. 2025 Aug 8;15(8):e71903. doi: 10.1002/ece3.71903 (PMC12332424; doi:10.1002/ece3.71903)
Supplement: Supplementary file 1 — Figure S1: ece371903‐sup‐0001‐FiguresS1.zip. [file ECE3-15-e71903-s005.zip › FigureS1/AppendixS1.docx]

**Supplemental Information for:**

**High Connectivity at Abyssal Depths: Genomic and Proteomic Insights into Population Structure of the Pan-Atlantic Deep-Sea Bivalve *Ledella ultima* (E. A. Smith, 1885)**

Jenny Neuhaus, Mark E. de Wilt, Sven Rossel, Saskia Brix, Ron J. Etter, Robert M. Jennings, Katrin Linse, Pedro Martínez Arbizu, Martin Schwentner, Janna Peters

**Table of Contents:**

| Figure S1 | Page 2 |
| --- | --- |
| Figure S2 | Page 3 |
| Figure S3 | Page 4 |
| Supplemental methods | Page 5 |
| Table S1 | Page 6 |
| Table S2 | Page 6 |
| Table S3 | Page 6 |
| References | Page 7 |


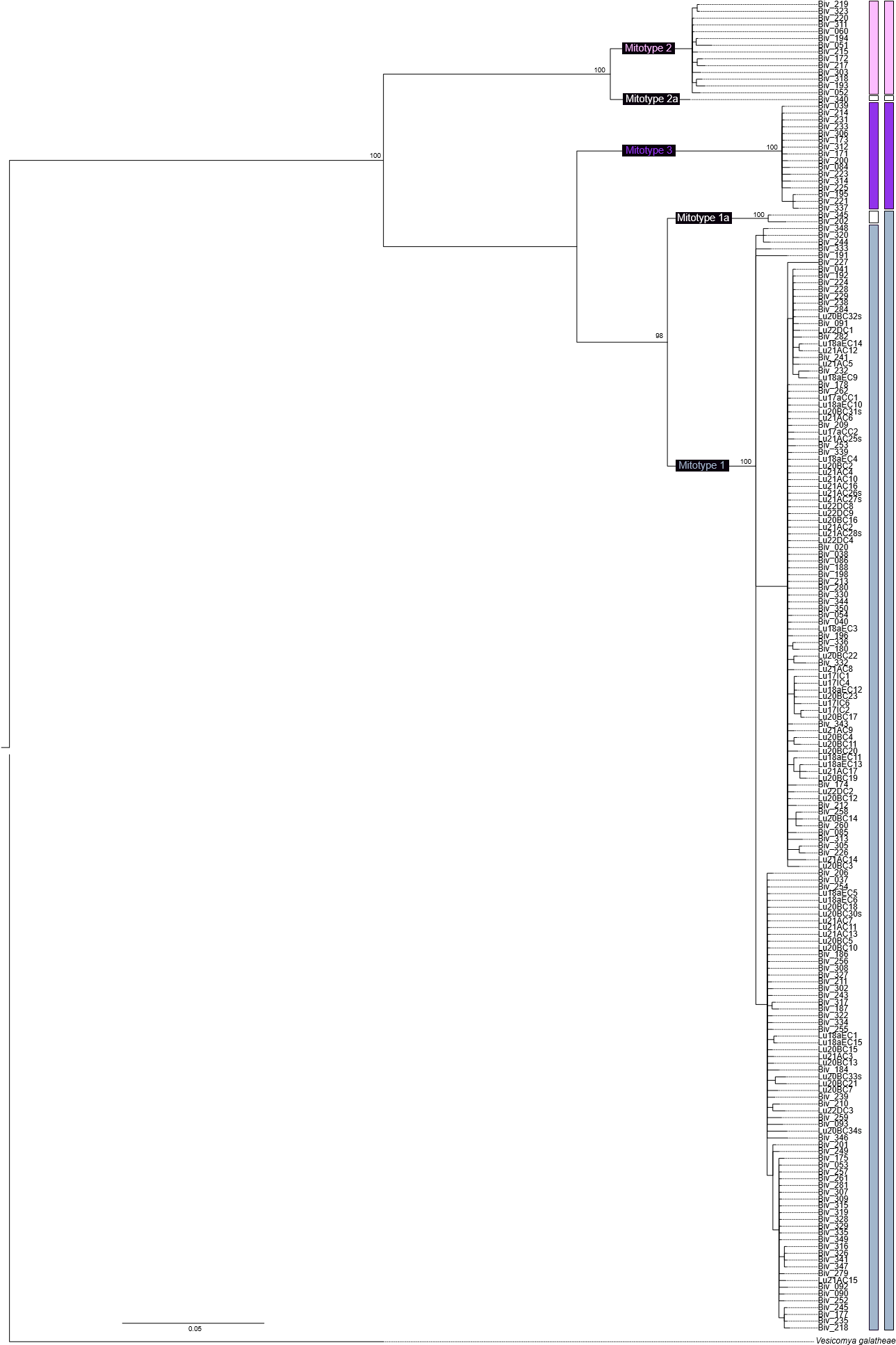


**Figure S1.** COI Bayesian tree with delimitation of mitotypes using the Assemble Species by Automatic Partitioning (ASAP) method. Colored bars visualize the results from the two best ASAP scores.


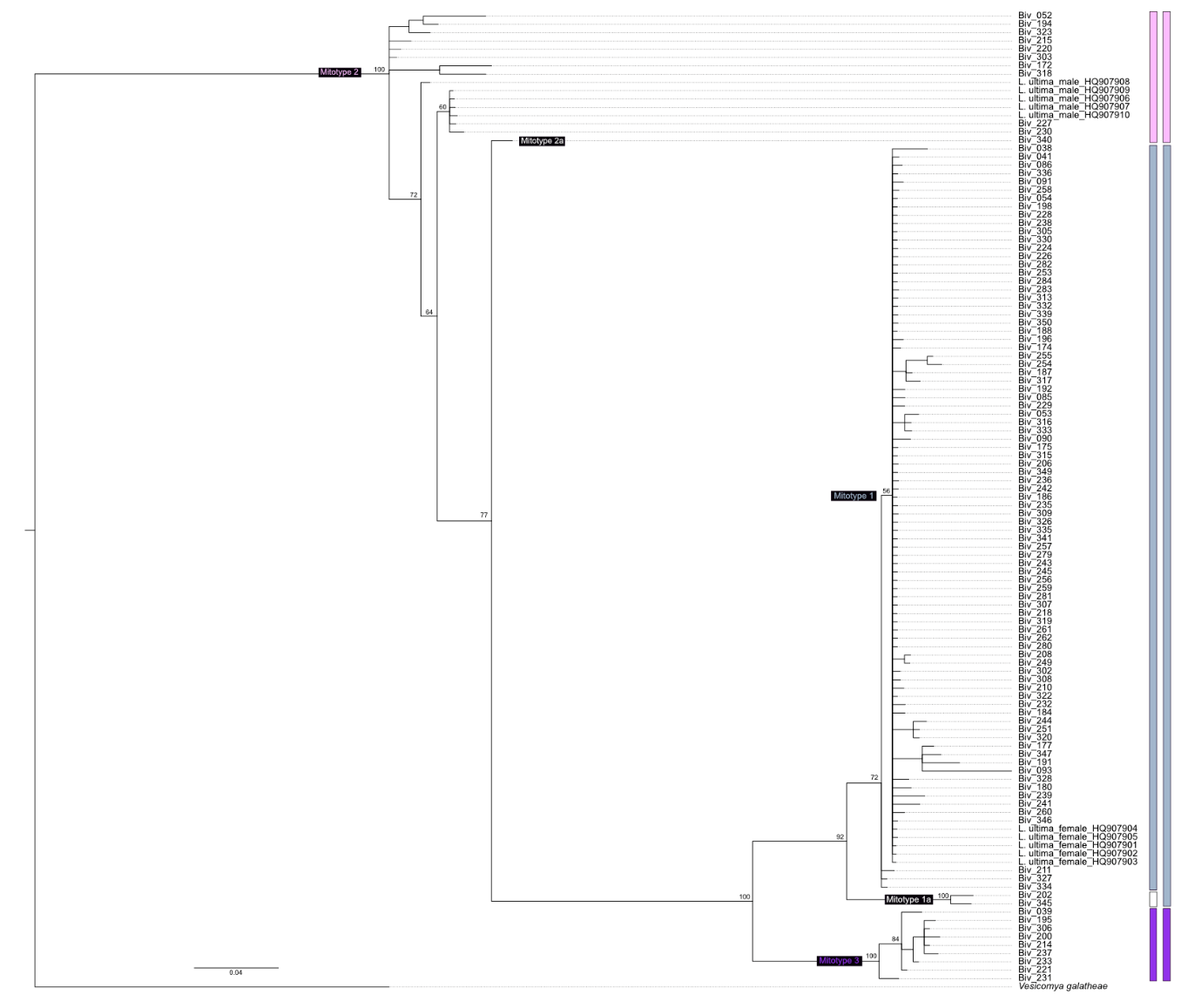


**Figure S2.** 16S Bayesian tree with delimitation of mitotypes using the Assemble Species by Automatic Partitioning (ASAP) method. Colored bars visualize the results from the two best ASAP scores.

**
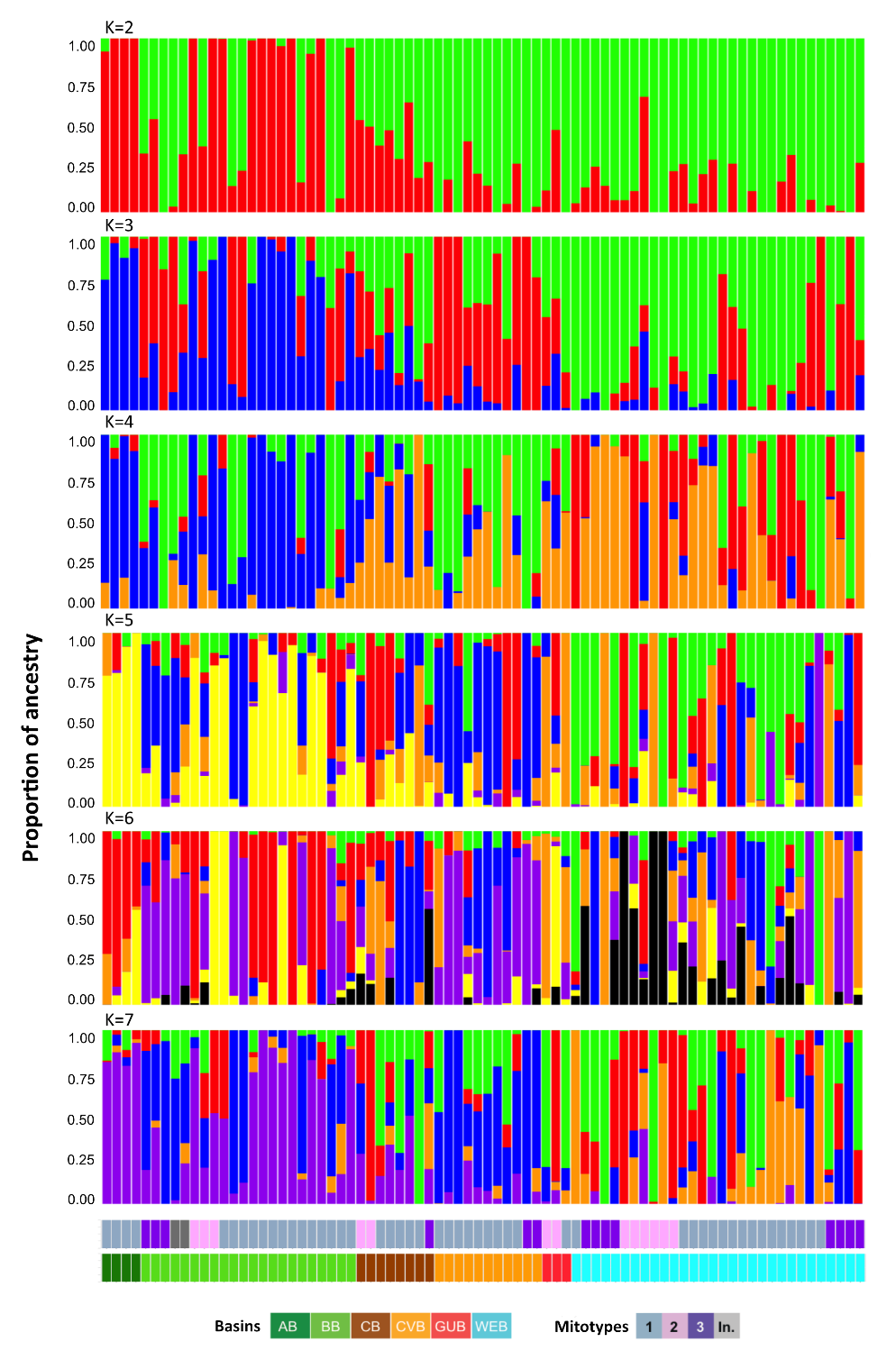
**

**Figure S3.** Patterns of population structure for *L. ultima* based on SNP data obtained by 2b-RAD sequencing. Proportions of ancestry for K = 2–7 using the Sparse Nonnegative Matrix Factorization (sNMF) algorithm. Colored bars below plots correspond to mitotypes (top) and six Atlantic basins (bottom). Abbreviations: AB: Argentine Basin, BB: Brazil Basin, CB: Cape Basin, CVB: Cape Verde Basin, GUB: Guyana Basin, WEB: West European Basin.

**Supplemental methods**

**COI sequence generation of female mitochondrial DNA listed in Table S1**

The genus *Ledella* is known to exhibit mitochondrial heteroplasmy in which the male mitochondrial genome, which is usually destroyed during embryonic development, is retained in adult males (Boyle & Etter, 2013). Male mitochondrial gene sequences are often highly divergent, but can still be amplified by universal PCR primers if those sites retain enough similarity. Therefore, to obtain only the female COI sequence from individuals of *L. ultima,* COI sequences from several individuals known to be male (via Boyle and Etter, 2013) were amplified with the universal primers of Folmer *et al.* (1994). PCRs were performed in 50µL reactions consisting of 1X GoTaq Flexi buffer with loading dye (Promega, WI, USA), 2.5 mM MgCl_2_, 2 pmol dNTPs, 1.2 pmol of each primer, 2µL genomic DNA, and 1 U of Taq polymerase (Promega). Conditions specific to each locus are given below. Reactions were run on a thermocycler with an initial denaturation at 94°C for 3 minutes, followed by 35 cycles of denaturation at 94°C for 30 seconds, annealing at 45°C for 45 seconds, and extension at 72°C for 1 minute. Final elongation took place at 72°C for 3 minutes with a final hold at 4°C. PCR products were purified with the Wizard SV Gel and PCR cleanup system (Promega) and cloned into *E. coli* using the pGEM-T vector system (Promega), following the manufacturer’s protocols. Individual clones were picked (*n* = 5 per male) and used directly as DNA template in PCR with an annealing temperature of 48°C, then sequenced with plasmid primers SP6 and T7 following the manufacturer’s protocol, and sequenced by Agencourt Inc. (Beckman−Coulter company, Beverly, MA, USA). Chromatograms were edited in Sequencher (Gene Codes Corp., Ann Arbor, MI, USA). Male and female COI sequences were aligned to closely-related protobranch COI sequences obtained from GenBank, using the CLUSTAL algorithm (Larkin et al., 2007) in BioEdit with default parameters. Female-specific PCR primers (forward LuFEMf1 5’-TTG GGC AGG TTT RAT AGG AAG TTA AGA TCA GGG AAA-3’; reverse LuFEMr1 5’-AAG ATC AGG GAA ATA ATG GAA TTT T-3’) were designed to amplify only the female COI sequence directly from genomic DNA extractions, employing an annealing temperature of 50°C. Successful PCRs were sequenced by Agencourt Inc. (Beckman−Coulter company, Beverly, MA, USA).

**Table S1**. List of additional sequences used to identify the occurrence of female and male mitochondrial DNA (mtDNA) amongst our sequenced specimens. Where applicable, GenBank accession numbers are provided. Of the specimens Lu20BC2–Lu20BC4, both the female and male mtDNA of 16S as well as the female mtDNA of COI were included in the data analysis. Respective assignments to mitotypes are given for each mitochondrial marker (COI, 16S) and sex-specific mtDNA. 16S sequences were obtained by Boyle and Etter (2013). Metadata for the specimens can be retrieved from Etter and Rex (2021).

see file “Table S1.xlsx”

**Table S2.** Collected specimens of *L. ultima* sorted by DNA voucher, with an outline of molecular methods applied on each specimen. Mitotype assignments correspond to haplotype networks. GenBank and SRA accession numbers are provided for mitochondrial DNA and 2b-RAD SNP data, respectively. Annotations: ^†^S = 2b-RAD samples excluded from analyses; x = Proteomic spectra included in analyses; SRA = NCBI Sequence Read Archive; SMF = Senckenberg Museum Frankfurt, Malacology Collection.

see file “Table S2.xlsx”

**Table S3.** Overview of loci, mean coverage per individual loci, total unfiltered SNPs, SNPs after filtering and pruning, and mean heterozygosity (HET) for stack depth m = 3, 5, 8.

see file “Table S3.xlxs”

**References**

Boyle, E. E., & Etter, R. J. (2013). Heteroplasmy in a deep-sea protobranch bivalve suggests an ancient origin of doubly uniparental inheritance of mitochondria in Bivalvia. *Marine Biology*, *160*, 413–422. https://doi.org/10.1007/s00227-012-2099-y

Etter, R. J., Boyle, E. E., Glazier, A., Jennings, R. M., Dutra, E., & Chase, M. R. (2011). Phylogeography of a pan-Atlantic abyssal protobranch bivalve: implications for evolution in the Deep Atlantic. *Molecular Ecology*, *20*, 829–843. https://doi.org/10.1111/j.1365-294X.2010.04978.x

Etter, R. J., & Rex, M. (2021). *Counts of Protobranch bivalves collected in a series of epibenthic sled samples taken on R/V Endeavor cruise EN447 in the Western North Atlantic (34-39N, 68-70W) in 2008 (ENAB project)*. Biological and Chemical Oceanography Data Management Office (BCO-DMO). https://doi.org/10.26008/1912/bco-dmo.542513.1

Folmer, O., Black, M., Hoeh, W., Lutz, R., & Vrijenhoek, R. (1994). DNA primers for amplification of mitochondrial cytochrome c oxidase subunit I from diverse metazoan invertebrates. *Molecular Marine Biology and Biotechnology*, *3*(5), 294–299. https://doi.org/10.1071/ZO9660275

Larkin, M. A., Blackshields, G., Brown, N. P., Chenna, R., Mcgettigan, P. A., McWilliam, H., Valentin, F., Wallace, I. M., Wilm, A., Lopez, R., Thompson, J. D., Gibson, T. J., & Higgins, D. G. (2007). Clustal W and Clustal X version 2.0. *Bioinformatics*, *23*(21), 2947–2948. https://doi.org/10.1093/bioinformatics/btm404
