# Supplementary figures and images for: High Connectivity at Abyssal Depths: Genomic and Proteomic Insights Into Population Structure of the Pan‐Atlantic Deep‐Sea Bivalve Ledella ultima (E. A. Smith, 1885)
Source: Ecol Evol. 2025 Aug 8;15(8):e71903. doi: 10.1002/ece3.71903 (PMC12332424; doi:10.1002/ece3.71903)

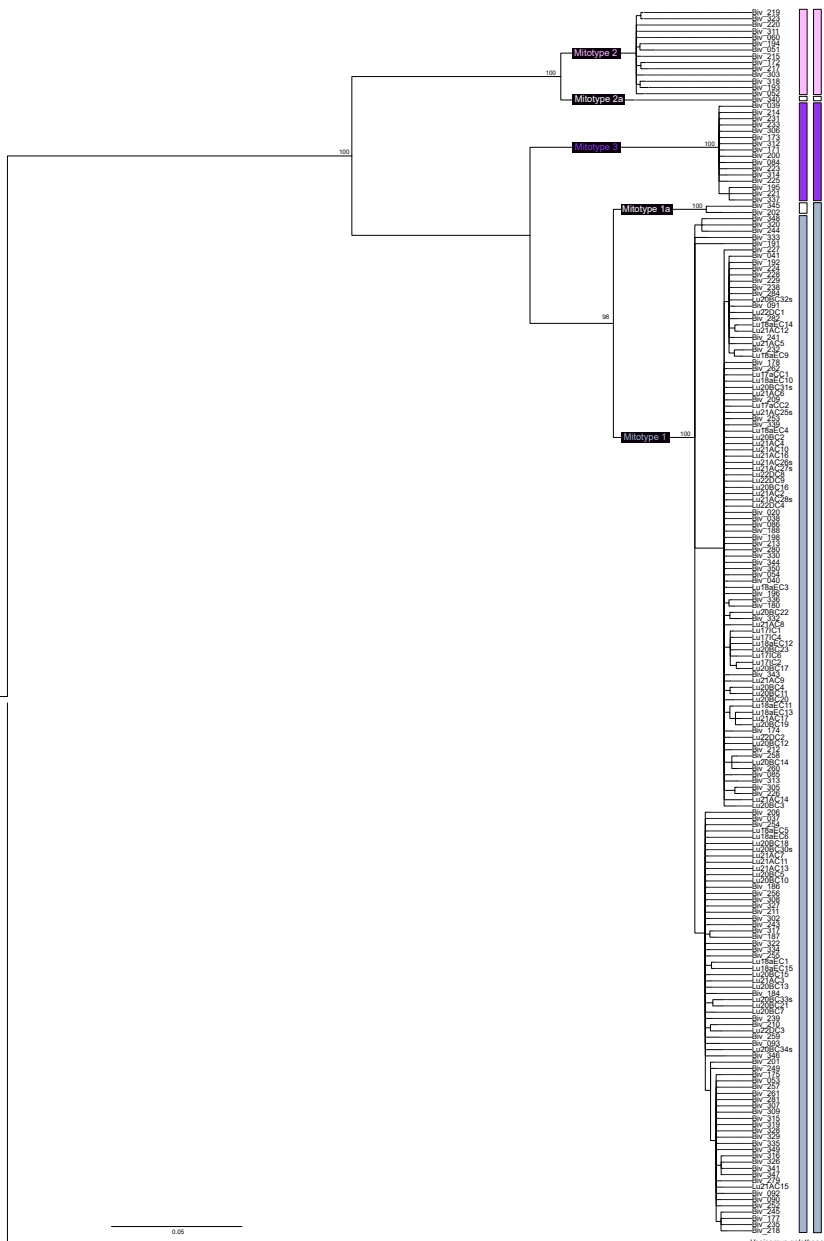

Supplement: Supplementary file 1 — Figure S1: ece371903‐sup‐0001‐FiguresS1.zip. [file ECE3-15-e71903-s005.zip › FigureS1/Figure S1.pdf]

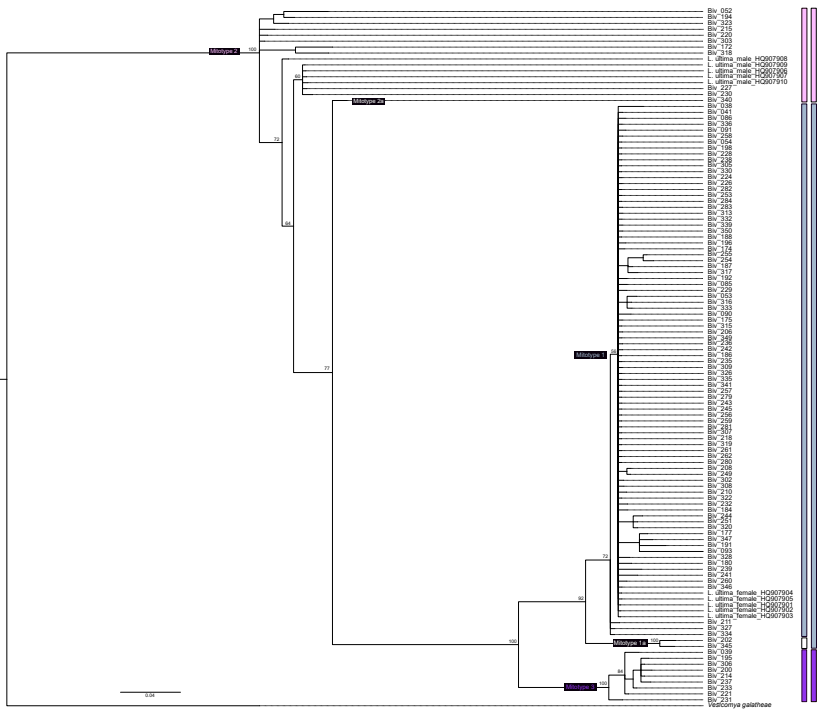

Supplement: Supplementary file 1 — Figure S1: ece371903‐sup‐0001‐FiguresS1.zip. [file ECE3-15-e71903-s005.zip › FigureS1/Figure S2.pdf]

K=2

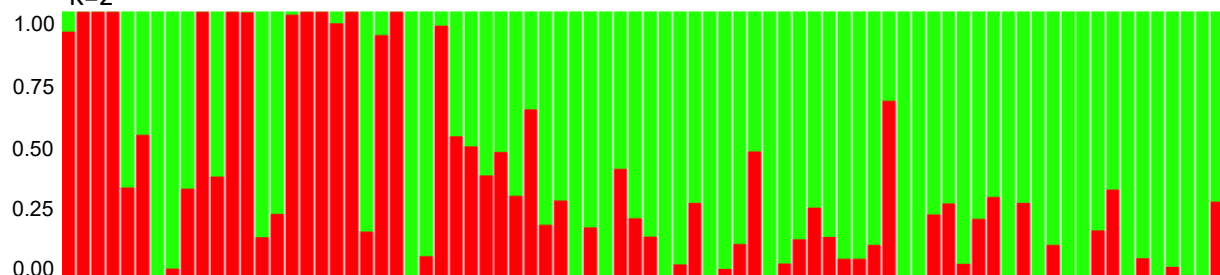

K=3

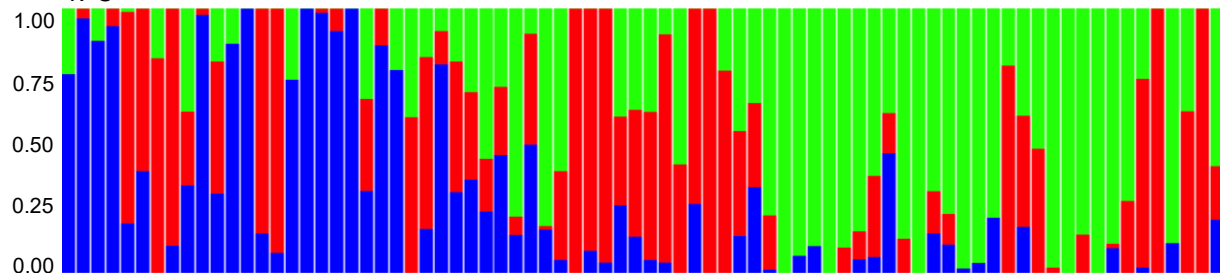

K=4

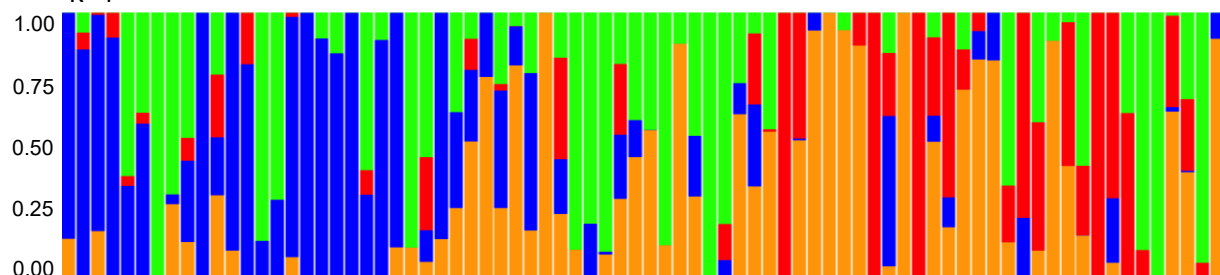

K=5

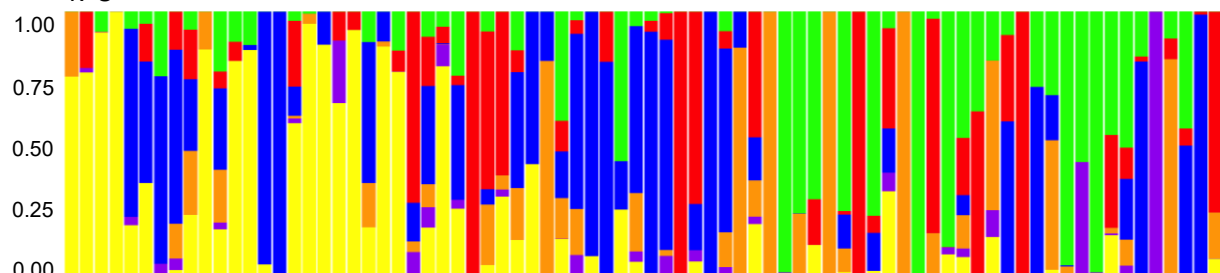

K=6

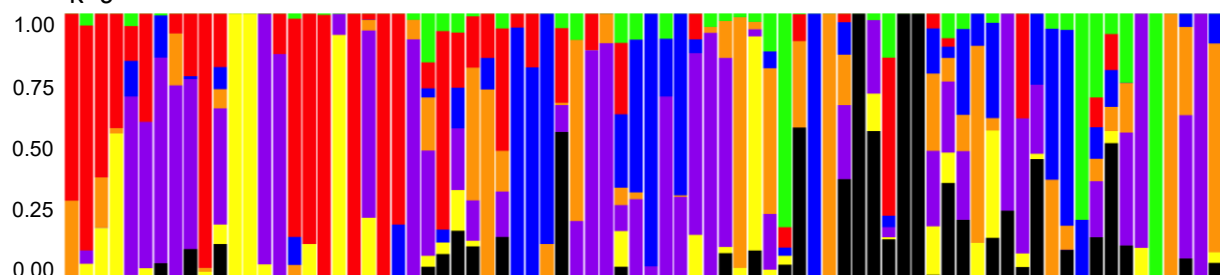

K=7

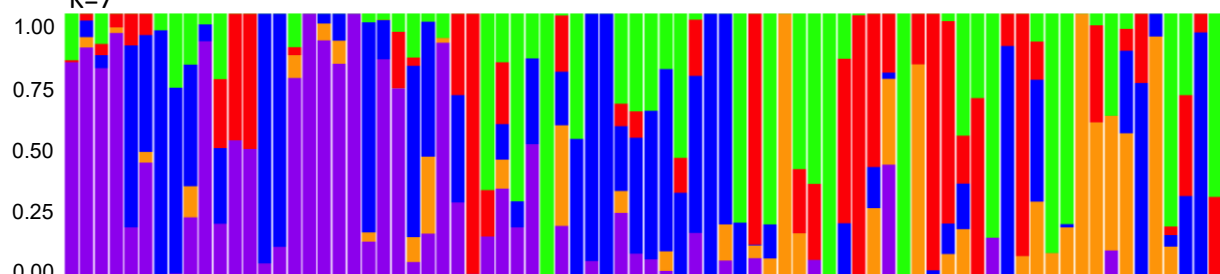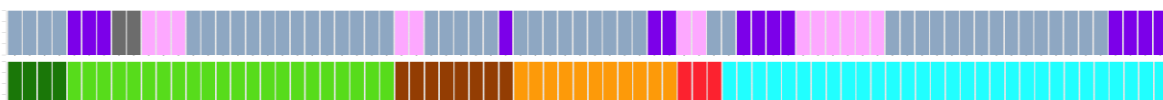

Basins

AB

BB

CB

CVB

GUB

WEB

Mitotypes

1

2

3

In.

Supplement: Supplementary file 1 — Figure S1: ece371903‐sup‐0001‐FiguresS1.zip. [file ECE3-15-e71903-s005.zip › FigureS1/Figure S3.pdf]
